# Measurement of jet shapes in top-quark pair events at $\sqrt{s} = 7 \ \mbox{TeV}$ using the ATLAS detector

**DOI:** 10.1140/epjc/s10052-013-2676-3

**Published:** 2013-12-11

**Authors:** The ATLAS Collaboration, G. Aad, T. Abajyan, B. Abbott, J. Abdallah, S. Abdel Khalek, A. A. Abdelalim, O. Abdinov, R. Aben, B. Abi, M. Abolins, O. S. AbouZeid, H. Abramowicz, H. Abreu, Y. Abulaiti, B. S. Acharya, L. Adamczyk, D. L. Adams, T. N. Addy, J. Adelman, S. Adomeit, T. Adye, S. Aefsky, T. Agatonovic-Jovin, J. A. Aguilar-Saavedra, M. Agustoni, S. P. Ahlen, F. Ahles, A. Ahmad, M. Ahsan, G. Aielli, T. P. A. Åkesson, G. Akimoto, A. V. Akimov, M. A. Alam, J. Albert, S. Albrand, M. J. Alconada Verzini, M. Aleksa, I. N. Aleksandrov, F. Alessandria, C. Alexa, G. Alexander, G. Alexandre, T. Alexopoulos, M. Alhroob, M. Aliev, G. Alimonti, J. Alison, B. M. M. Allbrooke, L. J. Allison, P. P. Allport, S. E. Allwood-Spiers, J. Almond, A. Aloisio, R. Alon, A. Alonso, F. Alonso, A. Altheimer, B. Alvarez Gonzalez, M. G. Alviggi, K. Amako, Y. Amaral Coutinho, C. Amelung, V. V. Ammosov, S. P. Amor Dos Santos, A. Amorim, S. Amoroso, N. Amram, C. Anastopoulos, L. S. Ancu, N. Andari, T. Andeen, C. F. Anders, G. Anders, K. J. Anderson, A. Andreazza, V. Andrei, X. S. Anduaga, S. Angelidakis, P. Anger, A. Angerami, F. Anghinolfi, A. V. Anisenkov, N. Anjos, A. Annovi, A. Antonaki, M. Antonelli, A. Antonov, J. Antos, F. Anulli, M. Aoki, L. Aperio Bella, R. Apolle, G. Arabidze, I. Aracena, Y. Arai, A. T. H. Arce, S. Arfaoui, J-F. Arguin, S. Argyropoulos, E. Arik, M. Arik, A. J. Armbruster, O. Arnaez, V. Arnal, A. Artamonov, G. Artoni, D. Arutinov, S. Asai, N. Asbah, S. Ask, B. Åsman, L. Asquith, K. Assamagan, R. Astalos, A. Astbury, M. Atkinson, B. Auerbach, E. Auge, K. Augsten, M. Aurousseau, G. Avolio, D. Axen, G. Azuelos, Y. Azuma, M. A. Baak, G. Baccaglioni, C. Bacci, A. M. Bach, H. Bachacou, K. Bachas, M. Backes, M. Backhaus, J. Backus Mayes, E. Badescu, P. Bagiacchi, P. Bagnaia, Y. Bai, D. C. Bailey, T. Bain, J. T. Baines, O. K. Baker, S. Baker, P. Balek, F. Balli, E. Banas, P. Banerjee, Sw. Banerjee, D. Banfi, A. Bangert, V. Bansal, H. S. Bansil, L. Barak, S. P. Baranov, T. Barber, E. L. Barberio, D. Barberis, M. Barbero, D. Y. Bardin, T. Barillari, M. Barisonzi, T. Barklow, N. Barlow, B. M. Barnett, R. M. Barnett, A. Baroncelli, G. Barone, A. J. Barr, F. Barreiro, J. Barreiro Guimarães da Costa, R. Bartoldus, A. E. Barton, V. Bartsch, A. Basye, R. L. Bates, L. Batkova, J. R. Batley, A. Battaglia, M. Battistin, F. Bauer, H. S. Bawa, S. Beale, T. Beau, P. H. Beauchemin, R. Beccherle, P. Bechtle, H. P. Beck, K. Becker, S. Becker, M. Beckingham, K. H. Becks, A. J. Beddall, A. Beddall, S. Bedikian, V. A. Bednyakov, C. P. Bee, L. J. Beemster, T. A. Beermann, M. Begel, C. Belanger-Champagne, P. J. Bell, W. H. Bell, G. Bella, L. Bellagamba, A. Bellerive, M. Bellomo, A. Belloni, O. L. Beloborodova, K. Belotskiy, O. Beltramello, O. Benary, D. Benchekroun, K. Bendtz, N. Benekos, Y. Benhammou, E. Benhar Noccioli, J. A. Benitez Garcia, D. P. Benjamin, J. R. Bensinger, K. Benslama, S. Bentvelsen, D. Berge, E. Bergeaas Kuutmann, N. Berger, F. Berghaus, E. Berglund, J. Beringer, P. Bernat, R. Bernhard, C. Bernius, F. U. Bernlochner, T. Berry, C. Bertella, F. Bertolucci, M. I. Besana, G. J. Besjes, N. Besson, S. Bethke, W. Bhimji, R. M. Bianchi, L. Bianchini, M. Bianco, O. Biebel, S. P. Bieniek, K. Bierwagen, J. Biesiada, M. Biglietti, H. Bilokon, M. Bindi, S. Binet, A. Bingul, C. Bini, B. Bittner, C. W. Black, J. E. Black, K. M. Black, D. Blackburn, R. E. Blair, J.-B. Blanchard, T. Blazek, I. Bloch, C. Blocker, J. Blocki, W. Blum, U. Blumenschein, G. J. Bobbink, V. S. Bobrovnikov, S. S. Bocchetta, A. Bocci, C. R. Boddy, M. Boehler, J. Boek, T. T. Boek, N. Boelaert, J. A. Bogaerts, A. G. Bogdanchikov, A. Bogouch, C. Bohm, J. Bohm, V. Boisvert, T. Bold, V. Boldea, N. M. Bolnet, M. Bomben, M. Bona, M. Boonekamp, S. Bordoni, C. Borer, A. Borisov, G. Borissov, M. Borri, S. Borroni, J. Bortfeldt, V. Bortolotto, K. Bos, D. Boscherini, M. Bosman, H. Boterenbrood, J. Bouchami, J. Boudreau, E. V. Bouhova-Thacker, D. Boumediene, C. Bourdarios, N. Bousson, S. Boutouil, A. Boveia, J. Boyd, I. R. Boyko, I. Bozovic-Jelisavcic, J. Bracinik, P. Branchini, A. Brandt, G. Brandt, O. Brandt, U. Bratzler, B. Brau, J. E. Brau, H. M. Braun, S. F. Brazzale, B. Brelier, J. Bremer, K. Brendlinger, R. Brenner, S. Bressler, T. M. Bristow, D. Britton, F. M. Brochu, I. Brock, R. Brock, F. Broggi, C. Bromberg, J. Bronner, G. Brooijmans, T. Brooks, W. K. Brooks, G. Brown, P. A. Bruckman de Renstrom, D. Bruncko, R. Bruneliere, S. Brunet, A. Bruni, G. Bruni, M. Bruschi, L. Bryngemark, T. Buanes, Q. Buat, F. Bucci, J. Buchanan, P. Buchholz, R. M. Buckingham, A. G. Buckley, S. I. Buda, I. A. Budagov, B. Budick, L. Bugge, O. Bulekov, A. C. Bundock, M. Bunse, T. Buran, H. Burckhart, S. Burdin, T. Burgess, S. Burke, E. Busato, V. Büscher, P. Bussey, C. P. Buszello, B. Butler, J. M. Butler, C. M. Buttar, J. M. Butterworth, W. Buttinger, M. Byszewski, S. Cabrera Urbán, D. Caforio, O. Cakir, P. Calafiura, G. Calderini, P. Calfayan, R. Calkins, L. P. Caloba, R. Caloi, D. Calvet, S. Calvet, R. Camacho Toro, P. Camarri, D. Cameron, L. M. Caminada, R. Caminal Armadans, S. Campana, M. Campanelli, V. Canale, F. Canelli, A. Canepa, J. Cantero, R. Cantrill, T. Cao, M. D. M. Capeans Garrido, I. Caprini, M. Caprini, D. Capriotti, M. Capua, R. Caputo, R. Cardarelli, T. Carli, G. Carlino, L. Carminati, S. Caron, E. Carquin, G. D. Carrillo-Montoya, A. A. Carter, J. R. Carter, J. Carvalho, D. Casadei, M. P. Casado, M. Cascella, C. Caso, E. Castaneda-Miranda, A. Castelli, V. Castillo Gimenez, N. F. Castro, G. Cataldi, P. Catastini, A. Catinaccio, J. R. Catmore, A. Cattai, G. Cattani, S. Caughron, V. Cavaliere, D. Cavalli, M. Cavalli-Sforza, V. Cavasinni, F. Ceradini, B. Cerio, A. S. Cerqueira, A. Cerri, L. Cerrito, F. Cerutti, A. Cervelli, S. A. Cetin, A. Chafaq, D. Chakraborty, I. Chalupkova, K. Chan, P. Chang, B. Chapleau, J. D. Chapman, J. W. Chapman, D. G. Charlton, V. Chavda, C. A. Chavez Barajas, S. Cheatham, S. Chekanov, S. V. Chekulaev, G. A. Chelkov, M. A. Chelstowska, C. Chen, H. Chen, S. Chen, X. Chen, Y. Chen, Y. Cheng, A. Cheplakov, R. Cherkaoui El Moursli, V. Chernyatin, E. Cheu, S. L. Cheung, L. Chevalier, V. Chiarella, G. Chiefari, J. T. Childers, A. Chilingarov, G. Chiodini, A. S. Chisholm, R. T. Chislett, A. Chitan, M. V. Chizhov, G. Choudalakis, S. Chouridou, B. K. B. Chow, I. A. Christidi, A. Christov, D. Chromek-Burckhart, M. L. Chu, J. Chudoba, G. Ciapetti, A. K. Ciftci, R. Ciftci, D. Cinca, V. Cindro, A. Ciocio, M. Cirilli, P. Cirkovic, Z. H. Citron, M. Citterio, M. Ciubancan, A. Clark, P. J. Clark, R. N. Clarke, J. C. Clemens, B. Clement, C. Clement, Y. Coadou, M. Cobal, A. Coccaro, J. Cochran, S. Coelli, L. Coffey, J. G. Cogan, J. Coggeshall, J. Colas, S. Cole, A. P. Colijn, N. J. Collins, C. Collins-Tooth, J. Collot, T. Colombo, G. Colon, G. Compostella, P. Conde Muiño, E. Coniavitis, M. C. Conidi, S. M. Consonni, V. Consorti, S. Constantinescu, C. Conta, G. Conti, F. Conventi, M. Cooke, B. D. Cooper, A. M. Cooper-Sarkar, N. J. Cooper-Smith, K. Copic, T. Cornelissen, M. Corradi, F. Corriveau, A. Corso-Radu, A. Cortes-Gonzalez, G. Cortiana, G. Costa, M. J. Costa, D. Costanzo, D. Côté, G. Cottin, L. Courneyea, G. Cowan, B. E. Cox, K. Cranmer, S. Crépé-Renaudin, F. Crescioli, M. Cristinziani, G. Crosetti, C.-M. Cuciuc, C. Cuenca Almenar, T. Cuhadar Donszelmann, J. Cummings, M. Curatolo, C. J. Curtis, C. Cuthbert, H. Czirr, P. Czodrowski, Z. Czyczula, S. D’Auria, M. D’Onofrio, A. D’Orazio, M. J. Da Cunha Sargedas De Sousa, C. Da Via, W. Dabrowski, A. Dafinca, T. Dai, F. Dallaire, C. Dallapiccola, M. Dam, D. S. Damiani, A. C. Daniells, H. O. Danielsson, V. Dao, G. Darbo, G. L. Darlea, S. Darmora, J. A. Dassoulas, W. Davey, T. Davidek, N. Davidson, E. Davies, M. Davies, O. Davignon, A. R. Davison, Y. Davygora, E. Dawe, I. Dawson, R. K. Daya-Ishmukhametova, K. De, R. de Asmundis, S. De Castro, S. De Cecco, J. de Graat, N. De Groot, P. de Jong, C. De La Taille, H. De la Torre, F. De Lorenzi, L. De Nooij, D. De Pedis, A. De Salvo, U. De Sanctis, A. De Santo, J. B. De Vivie De Regie, G. De Zorzi, W. J. Dearnaley, R. Debbe, C. Debenedetti, B. Dechenaux, D. V. Dedovich, J. Degenhardt, J. Del Peso, T. Del Prete, T. Delemontex, M. Deliyergiyev, A. Dell’Acqua, L. Dell’Asta, M. Della Pietra, D. della Volpe, M. Delmastro, P. A. Delsart, C. Deluca, S. Demers, M. Demichev, A. Demilly, B. Demirkoz, S. P. Denisov, D. Derendarz, J. E. Derkaoui, F. Derue, P. Dervan, K. Desch, P. O. Deviveiros, A. Dewhurst, B. DeWilde, S. Dhaliwal, R. Dhullipudi, A. Di Ciaccio, L. Di Ciaccio, C. Di Donato, A. Di Girolamo, B. Di Girolamo, S. Di Luise, A. Di Mattia, B. Di Micco, R. Di Nardo, A. Di Simone, R. Di Sipio, M. A. Diaz, E. B. Diehl, J. Dietrich, T. A. Dietzsch, S. Diglio, K. Dindar Yagci, J. Dingfelder, F. Dinut, C. Dionisi, P. Dita, S. Dita, F. Dittus, F. Djama, T. Djobava, M. A. B. do Vale, A. Do Valle Wemans, T. K. O. Doan, D. Dobos, E. Dobson, J. Dodd, C. Doglioni, T. Doherty, T. Dohmae, Y. Doi, J. Dolejsi, Z. Dolezal, B. A. Dolgoshein, M. Donadelli, J. Donini, J. Dopke, A. Doria, A. Dos Anjos, A. Dotti, M. T. Dova, A. T. Doyle, M. Dris, J. Dubbert, S. Dube, E. Dubreuil, E. Duchovni, G. Duckeck, D. Duda, A. Dudarev, F. Dudziak, L. Duflot, M-A. Dufour, L. Duguid, M. Dührssen, M. Dunford, H. Duran Yildiz, M. Düren, M. Dwuznik, J. Ebke, S. Eckweiler, W. Edson, C. A. Edwards, N. C. Edwards, W. Ehrenfeld, T. Eifert, G. Eigen, K. Einsweiler, E. Eisenhandler, T. Ekelof, M. El Kacimi, M. Ellert, S. Elles, F. Ellinghaus, K. Ellis, N. Ellis, J. Elmsheuser, M. Elsing, D. Emeliyanov, Y. Enari, O. C. Endner, R. Engelmann, A. Engl, J. Erdmann, A. Ereditato, D. Eriksson, J. Ernst, M. Ernst, J. Ernwein, D. Errede, S. Errede, E. Ertel, M. Escalier, H. Esch, C. Escobar, X. Espinal Curull, B. Esposito, F. Etienne, A. I. Etienvre, E. Etzion, D. Evangelakou, H. Evans, L. Fabbri, C. Fabre, G. Facini, R. M. Fakhrutdinov, S. Falciano, Y. Fang, M. Fanti, A. Farbin, A. Farilla, T. Farooque, S. Farrell, S. M. Farrington, P. Farthouat, F. Fassi, P. Fassnacht, D. Fassouliotis, B. Fatholahzadeh, A. Favareto, L. Fayard, P. Federic, O. L. Fedin, W. Fedorko, M. Fehling-Kaschek, L. Feligioni, C. Feng, E. J. Feng, H. Feng, A. B. Fenyuk, J. Ferencei, W. Fernando, S. Ferrag, J. Ferrando, V. Ferrara, A. Ferrari, P. Ferrari, R. Ferrari, D. E. Ferreira de Lima, A. Ferrer, D. Ferrere, C. Ferretti, A. Ferretto Parodi, M. Fiascaris, F. Fiedler, A. Filipčič, F. Filthaut, M. Fincke-Keeler, K. D. Finelli, M. C. N. Fiolhais, L. Fiorini, A. Firan, J. Fischer, M. J. Fisher, E. A. Fitzgerald, M. Flechl, I. Fleck, P. Fleischmann, S. Fleischmann, G. T. Fletcher, G. Fletcher, T. Flick, A. Floderus, L. R. Flores Castillo, A. C. Florez Bustos, M. J. Flowerdew, T. Fonseca Martin, A. Formica, A. Forti, D. Fortin, D. Fournier, H. Fox, P. Francavilla, M. Franchini, S. Franchino, D. Francis, M. Franklin, S. Franz, M. Fraternali, S. Fratina, S. T. French, C. Friedrich, F. Friedrich, D. Froidevaux, J. A. Frost, C. Fukunaga, E. Fullana Torregrosa, B. G. Fulsom, J. Fuster, C. Gabaldon, O. Gabizon, A. Gabrielli, A. Gabrielli, S. Gadatsch, T. Gadfort, S. Gadomski, G. Gagliardi, P. Gagnon, C. Galea, B. Galhardo, E. J. Gallas, V. Gallo, B. J. Gallop, P. Gallus, K. K. Gan, R. P. Gandrajula, Y. S. Gao, A. Gaponenko, F. M. Garay Walls, F. Garberson, C. García, J. E. García Navarro, M. Garcia-Sciveres, R. W. Gardner, N. Garelli, V. Garonne, C. Gatti, G. Gaudio, B. Gaur, L. Gauthier, P. Gauzzi, I. L. Gavrilenko, C. Gay, G. Gaycken, E. N. Gazis, P. Ge, Z. Gecse, C. N. P. Gee, D. A. A. Geerts, Ch. Geich-Gimbel, K. Gellerstedt, C. Gemme, A. Gemmell, M. H. Genest, S. Gentile, M. George, S. George, D. Gerbaudo, A. Gershon, H. Ghazlane, N. Ghodbane, B. Giacobbe, S. Giagu, V. Giangiobbe, P. Giannetti, F. Gianotti, B. Gibbard, A. Gibson, S. M. Gibson, M. Gilchriese, T. P. S. Gillam, D. Gillberg, A. R. Gillman, D. M. Gingrich, N. Giokaris, M. P. Giordani, R. Giordano, F. M. Giorgi, P. Giovannini, P. F. Giraud, D. Giugni, C. Giuliani, M. Giunta, B. K. Gjelsten, I. Gkialas, L. K. Gladilin, C. Glasman, J. Glatzer, A. Glazov, G. L. Glonti, M. Goblirsch-Kolb, J. R. Goddard, J. Godfrey, J. Godlewski, M. Goebel, C. Goeringer, S. Goldfarb, T. Golling, D. Golubkov, A. Gomes, L. S. Gomez Fajardo, R. Gonçalo, J. Goncalves Pinto Firmino Da Costa, L. Gonella, S. González de la Hoz, G. Gonzalez Parra, M. L. Gonzalez Silva, S. Gonzalez-Sevilla, J. J. Goodson, L. Goossens, P. A. Gorbounov, H. A. Gordon, I. Gorelov, G. Gorfine, B. Gorini, E. Gorini, A. Gorišek, E. Gornicki, A. T. Goshaw, C. Gössling, M. I. Gostkin, I. Gough Eschrich, M. Gouighri, D. Goujdami, M. P. Goulette, A. G. Goussiou, C. Goy, S. Gozpinar, L. Graber, I. Grabowska-Bold, P. Grafström, K-J. Grahn, E. Gramstad, F. Grancagnolo, S. Grancagnolo, V. Grassi, V. Gratchev, H. M. Gray, J. A. Gray, E. Graziani, O. G. Grebenyuk, T. Greenshaw, Z. D. Greenwood, K. Gregersen, I. M. Gregor, P. Grenier, J. Griffiths, N. Grigalashvili, A. A. Grillo, K. Grimm, S. Grinstein, Ph. Gris, Y. V. Grishkevich, J.-F. Grivaz, J. P. Grohs, A. Grohsjean, E. Gross, J. Grosse-Knetter, J. Groth-Jensen, K. Grybel, F. Guescini, D. Guest, O. Gueta, C. Guicheney, E. Guido, T. Guillemin, S. Guindon, U. Gul, J. Gunther, J. Guo, P. Gutierrez, N. Guttman, O. Gutzwiller, C. Guyot, C. Gwenlan, C. B. Gwilliam, A. Haas, S. Haas, C. Haber, H. K. Hadavand, P. Haefner, Z. Hajduk, H. Hakobyan, D. Hall, G. Halladjian, K. Hamacher, P. Hamal, K. Hamano, M. Hamer, A. Hamilton, S. Hamilton, L. Han, K. Hanagaki, K. Hanawa, M. Hance, C. Handel, P. Hanke, J. R. Hansen, J. B. Hansen, J. D. Hansen, P. H. Hansen, P. Hansson, K. Hara, A. S. Hard, T. Harenberg, S. Harkusha, D. Harper, R. D. Harrington, O. M. Harris, J. Hartert, F. Hartjes, T. Haruyama, A. Harvey, S. Hasegawa, Y. Hasegawa, S. Hassani, S. Haug, M. Hauschild, R. Hauser, M. Havranek, C. M. Hawkes, R. J. Hawkings, A. D. Hawkins, T. Hayakawa, T. Hayashi, D. Hayden, C. P. Hays, H. S. Hayward, S. J. Haywood, S. J. Head, T. Heck, V. Hedberg, L. Heelan, S. Heim, B. Heinemann, S. Heisterkamp, J. Hejbal, L. Helary, C. Heller, M. Heller, S. Hellman, D. Hellmich, C. Helsens, J. Henderson, R. C. W. Henderson, M. Henke, A. Henrichs, A. M. Henriques Correia, S. Henrot-Versille, C. Hensel, G. H. Herbert, C. M. Hernandez, Y. Hernández Jiménez, R. Herrberg-Schubert, G. Herten, R. Hertenberger, L. Hervas, G. G. Hesketh, N. P. Hessey, R. Hickling, E. Higón-Rodriguez, J. C. Hill, K. H. Hiller, S. Hillert, S. J. Hillier, I. Hinchliffe, E. Hines, M. Hirose, D. Hirschbuehl, J. Hobbs, N. Hod, M. C. Hodgkinson, P. Hodgson, A. Hoecker, M. R. Hoeferkamp, J. Hoffman, D. Hoffmann, J. I. Hofmann, M. Hohlfeld, S. O. Holmgren, J. L. Holzbauer, T. M. Hong, L. Hooft van Huysduynen, J-Y. Hostachy, S. Hou, A. Hoummada, J. Howard, J. Howarth, M. Hrabovsky, I. Hristova, J. Hrivnac, T. Hryn’ova, P. J. Hsu, S.-C. Hsu, D. Hu, X. Hu, Z. Hubacek, F. Hubaut, F. Huegging, A. Huettmann, T. B. Huffman, E. W. Hughes, G. Hughes, M. Huhtinen, T. A. Hülsing, M. Hurwitz, N. Huseynov, J. Huston, J. Huth, G. Iacobucci, G. Iakovidis, I. Ibragimov, L. Iconomidou-Fayard, J. Idarraga, P. Iengo, O. Igonkina, Y. Ikegami, K. Ikematsu, M. Ikeno, D. Iliadis, N. Ilic, T. Ince, P. Ioannou, M. Iodice, K. Iordanidou, V. Ippolito, A. Irles Quiles, C. Isaksson, M. Ishino, M. Ishitsuka, R. Ishmukhametov, C. Issever, S. Istin, A. V. Ivashin, W. Iwanski, H. Iwasaki, J. M. Izen, V. Izzo, B. Jackson, J. N. Jackson, P. Jackson, M. R. Jaekel, V. Jain, K. Jakobs, S. Jakobsen, T. Jakoubek, J. Jakubek, D. O. Jamin, D. K. Jana, E. Jansen, H. Jansen, J. Janssen, A. Jantsch, M. Janus, R. C. Jared, G. Jarlskog, L. Jeanty, G.-Y. Jeng, I. Jen-La Plante, D. Jennens, P. Jenni, J. Jentzsch, C. Jeske, P. Jež, S. Jézéquel, M. K. Jha, H. Ji, W. Ji, J. Jia, Y. Jiang, M. Jimenez Belenguer, S. Jin, O. Jinnouchi, M. D. Joergensen, D. Joffe, M. Johansen, K. E. Johansson, P. Johansson, S. Johnert, K. A. Johns, K. Jon-And, G. Jones, R. W. L. Jones, T. J. Jones, P. M. Jorge, K. D. Joshi, J. Jovicevic, X. Ju, C. A. Jung, R. M. Jungst, P. Jussel, A. Juste Rozas, S. Kabana, M. Kaci, A. Kaczmarska, P. Kadlecik, M. Kado, H. Kagan, M. Kagan, E. Kajomovitz, S. Kalinin, S. Kama, N. Kanaya, M. Kaneda, S. Kaneti, T. Kanno, V. A. Kantserov, J. Kanzaki, B. Kaplan, A. Kapliy, D. Kar, K. Karakostas, M. Karnevskiy, V. Kartvelishvili, A. N. Karyukhin, L. Kashif, G. Kasieczka, R. D. Kass, A. Kastanas, Y. Kataoka, J. Katzy, V. Kaushik, K. Kawagoe, T. Kawamoto, G. Kawamura, S. Kazama, V. F. Kazanin, M. Y. Kazarinov, R. Keeler, P. T. Keener, R. Kehoe, M. Keil, J. S. Keller, H. Keoshkerian, O. Kepka, B. P. Kerševan, S. Kersten, K. Kessoku, J. Keung, F. Khalil-zada, H. Khandanyan, A. Khanov, D. Kharchenko, A. Khodinov, A. Khomich, T. J. Khoo, G. Khoriauli, A. Khoroshilov, V. Khovanskiy, E. Khramov, J. Khubua, H. Kim, S. H. Kim, N. Kimura, O. Kind, B. T. King, M. King, R. S. B. King, S. B. King, J. Kirk, A. E. Kiryunin, T. Kishimoto, D. Kisielewska, T. Kitamura, T. Kittelmann, K. Kiuchi, E. Kladiva, M. Klein, U. Klein, K. Kleinknecht, M. Klemetti, A. Klier, P. Klimek, A. Klimentov, R. Klingenberg, J. A. Klinger, E. B. Klinkby, T. Klioutchnikova, P. F. Klok, E.-E. Kluge, P. Kluit, S. Kluth, E. Kneringer, E. B. F. G. Knoops, A. Knue, B. R. Ko, T. Kobayashi, M. Kobel, M. Kocian, P. Kodys, S. Koenig, F. Koetsveld, P. Koevesarki, T. Koffas, E. Koffeman, L. A. Kogan, S. Kohlmann, F. Kohn, Z. Kohout, T. Kohriki, T. Koi, H. Kolanoski, I. Koletsou, J. Koll, A. A. Komar, Y. Komori, T. Kondo, K. Köneke, A. C. König, T. Kono, A. I. Kononov, R. Konoplich, N. Konstantinidis, R. Kopeliansky, S. Koperny, L. Köpke, A. K. Kopp, K. Korcyl, K. Kordas, A. Korn, A. A. Korol, I. Korolkov, E. V. Korolkova, V. A. Korotkov, O. Kortner, S. Kortner, V. V. Kostyukhin, S. Kotov, V. M. Kotov, A. Kotwal, C. Kourkoumelis, V. Kouskoura, A. Koutsman, R. Kowalewski, T. Z. Kowalski, W. Kozanecki, A. S. Kozhin, V. Kral, V. A. Kramarenko, G. Kramberger, M. W. Krasny, A. Krasznahorkay, J. K. Kraus, A. Kravchenko, S. Kreiss, J. Kretzschmar, K. Kreutzfeldt, N. Krieger, P. Krieger, K. Kroeninger, H. Kroha, J. Kroll, J. Kroseberg, J. Krstic, U. Kruchonak, H. Krüger, T. Kruker, N. Krumnack, Z. V. Krumshteyn, A. Kruse, M. K. Kruse, T. Kubota, S. Kuday, S. Kuehn, A. Kugel, T. Kuhl, V. Kukhtin, Y. Kulchitsky, S. Kuleshov, M. Kuna, J. Kunkle, A. Kupco, H. Kurashige, M. Kurata, Y. A. Kurochkin, V. Kus, E. S. Kuwertz, M. Kuze, J. Kvita, R. Kwee, A. La Rosa, L. La Rotonda, L. Labarga, S. Lablak, C. Lacasta, F. Lacava, J. Lacey, H. Lacker, D. Lacour, V. R. Lacuesta, E. Ladygin, R. Lafaye, B. Laforge, T. Lagouri, S. Lai, H. Laier, E. Laisne, L. Lambourne, C. L. Lampen, W. Lampl, E. Lançon, U. Landgraf, M. P. J. Landon, V. S. Lang, C. Lange, A. J. Lankford, F. Lanni, K. Lantzsch, A. Lanza, S. Laplace, C. Lapoire, J. F. Laporte, T. Lari, A. Larner, M. Lassnig, P. Laurelli, V. Lavorini, W. Lavrijsen, P. Laycock, O. Le Dortz, E. Le Guirriec, E. Le Menedeu, T. LeCompte, F. Ledroit-Guillon, H. Lee, J. S. H. Lee, S. C. Lee, L. Lee, G. Lefebvre, M. Lefebvre, M. Legendre, F. Legger, C. Leggett, M. Lehmacher, G. Lehmann Miotto, A. G. Leister, M. A. L. Leite, R. Leitner, D. Lellouch, B. Lemmer, V. Lendermann, K. J. C. Leney, T. Lenz, G. Lenzen, B. Lenzi, K. Leonhardt, S. Leontsinis, F. Lepold, C. Leroy, J-R. Lessard, C. G. Lester, C. M. Lester, J. Levêque, D. Levin, L. J. Levinson, A. Lewis, G. H. Lewis, A. M. Leyko, M. Leyton, B. Li, B. Li, H. Li, H. L. Li, S. Li, X. Li, Z. Liang, H. Liao, B. Liberti, P. Lichard, K. Lie, J. Liebal, W. Liebig, C. Limbach, A. Limosani, M. Limper, S. C. Lin, F. Linde, B. E. Lindquist, J. T. Linnemann, E. Lipeles, A. Lipniacka, M. Lisovyi, T. M. Liss, D. Lissauer, A. Lister, A. M. Litke, D. Liu, J. B. Liu, K. Liu, L. Liu, M. Liu, M. Liu, Y. Liu, M. Livan, S. S. A. Livermore, A. Lleres, J. Llorente Merino, S. L. Lloyd, F. Lo Sterzo, E. Lobodzinska, P. Loch, W. S. Lockman, T. Loddenkoetter, F. K. Loebinger, A. E. Loevschall-Jensen, A. Loginov, C. W. Loh, T. Lohse, K. Lohwasser, M. Lokajicek, V. P. Lombardo, R. E. Long, L. Lopes, D. Lopez Mateos, J. Lorenz, N. Lorenzo Martinez, M. Losada, P. Loscutoff, M. J. Losty, X. Lou, A. Lounis, K. F. Loureiro, J. Love, P. A. Love, A. J. Lowe, F. Lu, H. J. Lubatti, C. Luci, A. Lucotte, D. Ludwig, I. Ludwig, J. Ludwig, F. Luehring, W. Lukas, L. Luminari, E. Lund, J. Lundberg, O. Lundberg, B. Lund-Jensen, J. Lundquist, M. Lungwitz, D. Lynn, R. Lysak, E. Lytken, H. Ma, L. L. Ma, G. Maccarrone, A. Macchiolo, B. Maček, J. Machado Miguens, D. Macina, R. Mackeprang, R. Madar, R. J. Madaras, H. J. Maddocks, W. F. Mader, A. Madsen, M. Maeno, T. Maeno, L. Magnoni, E. Magradze, K. Mahboubi, J. Mahlstedt, S. Mahmoud, G. Mahout, C. Maiani, C. Maidantchik, A. Maio, S. Majewski, Y. Makida, N. Makovec, P. Mal, B. Malaescu, Pa. Malecki, P. Malecki, V. P. Maleev, F. Malek, U. Mallik, D. Malon, C. Malone, S. Maltezos, V. M. Malyshev, S. Malyukov, J. Mamuzic, L. Mandelli, I. Mandić, R. Mandrysch, J. Maneira, A. Manfredini, L. Manhaes de Andrade Filho, J. A. Manjarres Ramos, A. Mann, P. M. Manning, A. Manousakis-Katsikakis, B. Mansoulie, R. Mantifel, L. Mapelli, L. March, J. F. Marchand, F. Marchese, G. Marchiori, M. Marcisovsky, C. P. Marino, C. N. Marques, F. Marroquim, Z. Marshall, L. F. Marti, S. Marti-Garcia, B. Martin, B. Martin, J. P. Martin, T. A. Martin, V. J. Martin, B. Martin dit Latour, H. Martinez, M. Martinez, S. Martin-Haugh, A. C. Martyniuk, M. Marx, F. Marzano, A. Marzin, L. Masetti, T. Mashimo, R. Mashinistov, J. Masik, A. L. Maslennikov, I. Massa, N. Massol, P. Mastrandrea, A. Mastroberardino, T. Masubuchi, H. Matsunaga, T. Matsushita, P. Mättig, S. Mättig, C. Mattravers, J. Maurer, S. J. Maxfield, D. A. Maximov, R. Mazini, M. Mazur, L. Mazzaferro, M. Mazzanti, S. P. Mc Kee, A. McCarn, R. L. McCarthy, T. G. McCarthy, N. A. McCubbin, K. W. McFarlane, J. A. Mcfayden, G. Mchedlidze, T. Mclaughlan, S. J. McMahon, R. A. McPherson, A. Meade, J. Mechnich, M. Mechtel, M. Medinnis, S. Meehan, R. Meera-Lebbai, T. Meguro, S. Mehlhase, A. Mehta, K. Meier, C. Meineck, B. Meirose, C. Melachrinos, B. R. Mellado Garcia, F. Meloni, L. Mendoza Navas, A. Mengarelli, S. Menke, E. Meoni, K. M. Mercurio, N. Meric, P. Mermod, L. Merola, C. Meroni, F. S. Merritt, H. Merritt, A. Messina, J. Metcalfe, A. S. Mete, C. Meyer, C. Meyer, J-P. Meyer, J. Meyer, J. Meyer, S. Michal, R. P. Middleton, S. Migas, L. Mijović, G. Mikenberg, M. Mikestikova, M. Mikuž, D. W. Miller, W. J. Mills, C. Mills, A. Milov, D. A. Milstead, D. Milstein, A. A. Minaenko, M. Miñano Moya, I. A. Minashvili, A. I. Mincer, B. Mindur, M. Mineev, Y. Ming, L. M. Mir, G. Mirabelli, J. Mitrevski, V. A. Mitsou, S. Mitsui, P. S. Miyagawa, J. U. Mjörnmark, T. Moa, V. Moeller, S. Mohapatra, W. Mohr, R. Moles-Valls, A. Molfetas, K. Mönig, C. Monini, J. Monk, E. Monnier, J. Montejo Berlingen, F. Monticelli, S. Monzani, R. W. Moore, C. Mora Herrera, A. Moraes, N. Morange, J. Morel, D. Moreno, M. Moreno Llácer, P. Morettini, M. Morgenstern, M. Morii, S. Moritz, A. K. Morley, G. Mornacchi, J. D. Morris, L. Morvaj, N. Möser, H. G. Moser, M. Mosidze, J. Moss, R. Mount, E. Mountricha, S. V. Mouraviev, E. J. W. Moyse, R. D. Mudd, F. Mueller, J. Mueller, K. Mueller, T. Mueller, T. Mueller, D. Muenstermann, Y. Munwes, J. A. Murillo Quijada, W. J. Murray, I. Mussche, E. Musto, A. G. Myagkov, M. Myska, O. Nackenhorst, J. Nadal, K. Nagai, R. Nagai, Y. Nagai, K. Nagano, A. Nagarkar, Y. Nagasaka, M. Nagel, A. M. Nairz, Y. Nakahama, K. Nakamura, T. Nakamura, I. Nakano, H. Namasivayam, G. Nanava, A. Napier, R. Narayan, M. Nash, T. Nattermann, T. Naumann, G. Navarro, H. A. Neal, P. Yu. Nechaeva, T. J. Neep, A. Negri, G. Negri, M. Negrini, S. Nektarijevic, A. Nelson, T. K. Nelson, S. Nemecek, P. Nemethy, A. A. Nepomuceno, M. Nessi, M. S. Neubauer, M. Neumann, A. Neusiedl, R. M. Neves, P. Nevski, F. M. Newcomer, P. R. Newman, D. H. Nguyen, V. Nguyen Thi Hong, R. B. Nickerson, R. Nicolaidou, B. Nicquevert, F. Niedercorn, J. Nielsen, N. Nikiforou, A. Nikiforov, V. Nikolaenko, I. Nikolic-Audit, K. Nikolics, K. Nikolopoulos, P. Nilsson, Y. Ninomiya, A. Nisati, R. Nisius, T. Nobe, L. Nodulman, M. Nomachi, I. Nomidis, S. Norberg, M. Nordberg, J. Novakova, M. Nozaki, L. Nozka, A.-E. Nuncio-Quiroz, G. Nunes Hanninger, T. Nunnemann, E. Nurse, B. J. O’Brien, D. C. O’Neil, V. O’Shea, L. B. Oakes, F. G. Oakham, H. Oberlack, J. Ocariz, A. Ochi, M. I. Ochoa, S. Oda, S. Odaka, J. Odier, H. Ogren, A. Oh, S. H. Oh, C. C. Ohm, T. Ohshima, W. Okamura, H. Okawa, Y. Okumura, T. Okuyama, A. Olariu, A. G. Olchevski, S. A. Olivares Pino, M. Oliveira, D. Oliveira Damazio, E. Oliver Garcia, D. Olivito, A. Olszewski, J. Olszowska, A. Onofre, P. U. E. Onyisi, C. J. Oram, M. J. Oreglia, Y. Oren, D. Orestano, N. Orlando, C. Oropeza Barrera, R. S. Orr, B. Osculati, R. Ospanov, G. Otero y Garzon, J. P. Ottersbach, M. Ouchrif, E. A. Ouellette, F. Ould-Saada, A. Ouraou, Q. Ouyang, A. Ovcharova, M. Owen, S. Owen, V. E. Ozcan, N. Ozturk, A. Pacheco Pages, C. Padilla Aranda, S. Pagan Griso, E. Paganis, C. Pahl, F. Paige, P. Pais, K. Pajchel, G. Palacino, C. P. Paleari, S. Palestini, D. Pallin, A. Palma, J. D. Palmer, Y. B. Pan, E. Panagiotopoulou, J. G. Panduro Vazquez, P. Pani, N. Panikashvili, S. Panitkin, D. Pantea, A. Papadelis, Th. D. Papadopoulou, K. Papageorgiou, A. Paramonov, D. Paredes Hernandez, W. Park, M. A. Parker, F. Parodi, J. A. Parsons, U. Parzefall, S. Pashapour, E. Pasqualucci, S. Passaggio, A. Passeri, F. Pastore, Fr. Pastore, G. Pásztor, S. Pataraia, N. D. Patel, J. R. Pater, S. Patricelli, T. Pauly, J. Pearce, M. Pedersen, S. Pedraza Lopez, M. I. Pedraza Morales, S. V. Peleganchuk, D. Pelikan, H. Peng, B. Penning, A. Penson, J. Penwell, T. Perez Cavalcanti, E. Perez Codina, M. T. Pérez García-Estañ, V. Perez Reale, L. Perini, H. Pernegger, R. Perrino, P. Perrodo, V. D. Peshekhonov, K. Peters, R. F. Y. Peters, B. A. Petersen, J. Petersen, T. C. Petersen, E. Petit, A. Petridis, C. Petridou, E. Petrolo, F. Petrucci, D. Petschull, M. Petteni, R. Pezoa, A. Phan, P. W. Phillips, G. Piacquadio, E. Pianori, A. Picazio, E. Piccaro, M. Piccinini, S. M. Piec, R. Piegaia, D. T. Pignotti, J. E. Pilcher, A. D. Pilkington, J. Pina, M. Pinamonti, A. Pinder, J. L. Pinfold, A. Pingel, B. Pinto, C. Pizio, M.-A. Pleier, V. Pleskot, E. Plotnikova, P. Plucinski, S. Poddar, F. Podlyski, R. Poettgen, L. Poggioli, D. Pohl, M. Pohl, G. Polesello, A. Policicchio, R. Polifka, A. Polini, V. Polychronakos, D. Pomeroy, K. Pommès, L. Pontecorvo, B. G. Pope, G. A. Popeneciu, D. S. Popovic, A. Poppleton, X. Portell Bueso, G. E. Pospelov, S. Pospisil, I. N. Potrap, C. J. Potter, C. T. Potter, G. Poulard, J. Poveda, V. Pozdnyakov, R. Prabhu, P. Pralavorio, A. Pranko, S. Prasad, R. Pravahan, S. Prell, K. Pretzl, D. Price, J. Price, L. E. Price, D. Prieur, M. Primavera, M. Proissl, K. Prokofiev, F. Prokoshin, E. Protopapadaki, S. Protopopescu, J. Proudfoot, X. Prudent, M. Przybycien, H. Przysiezniak, S. Psoroulas, E. Ptacek, E. Pueschel, D. Puldon, M. Purohit, P. Puzo, Y. Pylypchenko, J. Qian, A. Quadt, D. R. Quarrie, W. B. Quayle, D. Quilty, M. Raas, V. Radeka, V. Radescu, P. Radloff, F. Ragusa, G. Rahal, S. Rajagopalan, M. Rammensee, M. Rammes, A. S. Randle-Conde, K. Randrianarivony, C. Rangel-Smith, K. Rao, F. Rauscher, T. C. Rave, T. Ravenscroft, M. Raymond, A. L. Read, D. M. Rebuzzi, A. Redelbach, G. Redlinger, R. Reece, K. Reeves, A. Reinsch, I. Reisinger, M. Relich, C. Rembser, Z. L. Ren, A. Renaud, M. Rescigno, S. Resconi, B. Resende, P. Reznicek, R. Rezvani, R. Richter, E. Richter-Was, M. Ridel, P. Rieck, M. Rijssenbeek, A. Rimoldi, L. Rinaldi, R. R. Rios, E. Ritsch, I. Riu, G. Rivoltella, F. Rizatdinova, E. Rizvi, S. H. Robertson, A. Robichaud-Veronneau, D. Robinson, J. E. M. Robinson, A. Robson, J. G. Rocha de Lima, C. Roda, D. Roda Dos Santos, A. Roe, S. Roe, O. Røhne, S. Rolli, A. Romaniouk, M. Romano, G. Romeo, E. Romero Adam, N. Rompotis, L. Roos, E. Ros, S. Rosati, K. Rosbach, A. Rose, M. Rose, G. A. Rosenbaum, P. L. Rosendahl, O. Rosenthal, V. Rossetti, E. Rossi, L. P. Rossi, M. Rotaru, I. Roth, J. Rothberg, D. Rousseau, C. R. Royon, A. Rozanov, Y. Rozen, X. Ruan, F. Rubbo, I. Rubinskiy, N. Ruckstuhl, V. I. Rud, C. Rudolph, M. S. Rudolph, F. Rühr, A. Ruiz-Martinez, L. Rumyantsev, Z. Rurikova, N. A. Rusakovich, A. Ruschke, J. P. Rutherfoord, N. Ruthmann, P. Ruzicka, Y. F. Ryabov, M. Rybar, G. Rybkin, N. C. Ryder, A. F. Saavedra, A. Saddique, I. Sadeh, H. F-W. Sadrozinski, R. Sadykov, F. Safai Tehrani, H. Sakamoto, G. Salamanna, A. Salamon, M. Saleem, D. Salek, D. Salihagic, A. Salnikov, J. Salt, B. M. Salvachua Ferrando, D. Salvatore, F. Salvatore, A. Salvucci, A. Salzburger, D. Sampsonidis, A. Sanchez, J. Sánchez, V. Sanchez Martinez, H. Sandaker, H. G. Sander, M. P. Sanders, M. Sandhoff, T. Sandoval, C. Sandoval, R. Sandstroem, D. P. C. Sankey, A. Sansoni, C. Santoni, R. Santonico, H. Santos, I. Santoyo Castillo, K. Sapp, J. G. Saraiva, T. Sarangi, E. Sarkisyan-Grinbaum, B. Sarrazin, F. Sarri, G. Sartisohn, O. Sasaki, Y. Sasaki, N. Sasao, I. Satsounkevitch, G. Sauvage, E. Sauvan, J. B. Sauvan, P. Savard, V. Savinov, D. O. Savu, C. Sawyer, L. Sawyer, D. H. Saxon, J. Saxon, C. Sbarra, A. Sbrizzi, D. A. Scannicchio, M. Scarcella, J. Schaarschmidt, P. Schacht, D. Schaefer, A. Schaelicke, S. Schaepe, S. Schaetzel, U. Schäfer, A. C. Schaffer, D. Schaile, R. D. Schamberger, V. Scharf, V. A. Schegelsky, D. Scheirich, M. Schernau, M. I. Scherzer, C. Schiavi, J. Schieck, C. Schillo, M. Schioppa, S. Schlenker, E. Schmidt, K. Schmieden, C. Schmitt, C. Schmitt, S. Schmitt, B. Schneider, Y. J. Schnellbach, U. Schnoor, L. Schoeffel, A. Schoening, A. L. S. Schorlemmer, M. Schott, D. Schouten, J. Schovancova, M. Schram, C. Schroeder, N. Schroer, M. J. Schultens, H.-C. Schultz-Coulon, H. Schulz, M. Schumacher, B. A. Schumm, Ph. Schune, A. Schwartzman, Ph. Schwegler, Ph. Schwemling, R. Schwienhorst, J. Schwindling, T. Schwindt, M. Schwoerer, F. G. Sciacca, E. Scifo, G. Sciolla, W. G. Scott, F. Scutti, J. Searcy, G. Sedov, E. Sedykh, S. C. Seidel, A. Seiden, F. Seifert, J. M. Seixas, G. Sekhniaidze, S. J. Sekula, K. E. Selbach, D. M. Seliverstov, G. Sellers, M. Seman, N. Semprini-Cesari, C. Serfon, L. Serin, L. Serkin, T. Serre, R. Seuster, H. Severini, A. Sfyrla, E. Shabalina, M. Shamim, L. Y. Shan, J. T. Shank, Q. T. Shao, M. Shapiro, P. B. Shatalov, K. Shaw, P. Sherwood, S. Shimizu, M. Shimojima, T. Shin, M. Shiyakova, A. Shmeleva, M. J. Shochet, D. Short, S. Shrestha, E. Shulga, M. A. Shupe, P. Sicho, A. Sidoti, F. Siegert, Dj. Sijacki, O. Silbert, J. Silva, Y. Silver, D. Silverstein, S. B. Silverstein, V. Simak, O. Simard, Lj. Simic, S. Simion, E. Simioni, B. Simmons, R. Simoniello, M. Simonyan, P. Sinervo, N. B. Sinev, V. Sipica, G. Siragusa, A. Sircar, A. N. Sisakyan, S. Yu. Sivoklokov, J. Sjölin, T. B. Sjursen, L. A. Skinnari, H. P. Skottowe, K. Yu. Skovpen, P. Skubic, M. Slater, T. Slavicek, K. Sliwa, V. Smakhtin, B. H. Smart, L. Smestad, S. Yu. Smirnov, Y. Smirnov, L. N. Smirnova, O. Smirnova, K. M. Smith, M. Smizanska, K. Smolek, A. A. Snesarev, G. Snidero, J. Snow, S. Snyder, R. Sobie, J. Sodomka, A. Soffer, D. A. Soh, C. A. Solans, M. Solar, J. Solc, E. Yu. Soldatov, U. Soldevila, E. Solfaroli Camillocci, A. A. Solodkov, O. V. Solovyanov, V. Solovyev, N. Soni, A. Sood, V. Sopko, B. Sopko, M. Sosebee, R. Soualah, P. Soueid, A. M. Soukharev, D. South, S. Spagnolo, F. Spanò, R. Spighi, G. Spigo, R. Spiwoks, M. Spousta, T. Spreitzer, B. Spurlock, R. D. St. Denis, J. Stahlman, R. Stamen, E. Stanecka, R. W. Stanek, C. Stanescu, M. Stanescu-Bellu, M. M. Stanitzki, S. Stapnes, E. A. Starchenko, J. Stark, P. Staroba, P. Starovoitov, R. Staszewski, A. Staude, P. Stavina, G. Steele, P. Steinbach, P. Steinberg, I. Stekl, B. Stelzer, H. J. Stelzer, O. Stelzer-Chilton, H. Stenzel, S. Stern, G. A. Stewart, J. A. Stillings, M. C. Stockton, M. Stoebe, K. Stoerig, G. Stoicea, S. Stonjek, A. R. Stradling, A. Straessner, J. Strandberg, S. Strandberg, A. Strandlie, M. Strang, E. Strauss, M. Strauss, P. Strizenec, R. Ströhmer, D. M. Strom, J. A. Strong, R. Stroynowski, B. Stugu, I. Stumer, J. Stupak, P. Sturm, N. A. Styles, D. Su, HS. Subramania, R. Subramaniam, A. Succurro, Y. Sugaya, C. Suhr, M. Suk, V. V. Sulin, S. Sultansoy, T. Sumida, X. Sun, J. E. Sundermann, K. Suruliz, G. Susinno, M. R. Sutton, Y. Suzuki, Y. Suzuki, M. Svatos, S. Swedish, M. Swiatlowski, I. Sykora, T. Sykora, D. Ta, K. Tackmann, A. Taffard, R. Tafirout, N. Taiblum, Y. Takahashi, H. Takai, R. Takashima, H. Takeda, T. Takeshita, Y. Takubo, M. Talby, A. A. Talyshev, J. Y. C. Tam, M. C. Tamsett, K. G. Tan, J. Tanaka, R. Tanaka, S. Tanaka, S. Tanaka, A. J. Tanasijczuk, K. Tani, N. Tannoury, S. Tapprogge, S. Tarem, F. Tarrade, G. F. Tartarelli, P. Tas, M. Tasevsky, T. Tashiro, E. Tassi, Y. Tayalati, C. Taylor, F. E. Taylor, G. N. Taylor, W. Taylor, M. Teinturier, F. A. Teischinger, M. Teixeira Dias Castanheira, P. Teixeira-Dias, K. K. Temming, H. Ten Kate, P. K. Teng, S. Terada, K. Terashi, J. Terron, M. Testa, R. J. Teuscher, J. Therhaag, T. Theveneaux-Pelzer, S. Thoma, J. P. Thomas, E. N. Thompson, P. D. Thompson, P. D. Thompson, A. S. Thompson, L. A. Thomsen, E. Thomson, M. Thomson, W. M. Thong, R. P. Thun, F. Tian, M. J. Tibbetts, T. Tic, V. O. Tikhomirov, Yu. A. Tikhonov, S. Timoshenko, E. Tiouchichine, P. Tipton, S. Tisserant, T. Todorov, S. Todorova-Nova, B. Toggerson, J. Tojo, S. Tokár, K. Tokushuku, K. Tollefson, L. Tomlinson, M. Tomoto, L. Tompkins, K. Toms, A. Tonoyan, C. Topfel, N. D. Topilin, E. Torrence, H. Torres, E. Torró Pastor, J. Toth, F. Touchard, D. R. Tovey, H. L. Tran, T. Trefzger, L. Tremblet, A. Tricoli, I. M. Trigger, S. Trincaz-Duvoid, M. F. Tripiana, N. Triplett, W. Trischuk, B. Trocmé, C. Troncon, M. Trottier-McDonald, M. Trovatelli, P. True, M. Trzebinski, A. Trzupek, C. Tsarouchas, J. C-L. Tseng, M. Tsiakiris, P. V. Tsiareshka, D. Tsionou, G. Tsipolitis, S. Tsiskaridze, V. Tsiskaridze, E. G. Tskhadadze, I. I. Tsukerman, V. Tsulaia, J.-W. Tsung, S. Tsuno, D. Tsybychev, A. Tua, A. Tudorache, V. Tudorache, J. M. Tuggle, A. N. Tuna, M. Turala, D. Turecek, I. Turk Cakir, R. Turra, P. M. Tuts, A. Tykhonov, M. Tylmad, M. Tyndel, K. Uchida, I. Ueda, R. Ueno, M. Ughetto, M. Ugland, M. Uhlenbrock, F. Ukegawa, G. Unal, A. Undrus, G. Unel, F. C. Ungaro, Y. Unno, D. Urbaniec, P. Urquijo, G. Usai, L. Vacavant, V. Vacek, B. Vachon, S. Vahsen, N. Valencic, S. Valentinetti, A. Valero, L. Valery, S. Valkar, E. Valladolid Gallego, S. Vallecorsa, J. A. Valls Ferrer, R. Van Berg, P. C. Van Der Deijl, R. van der Geer, H. van der Graaf, R. Van Der Leeuw, D. van der Ster, N. van Eldik, P. van Gemmeren, J. Van Nieuwkoop, I. van Vulpen, M. Vanadia, W. Vandelli, A. Vaniachine, P. Vankov, F. Vannucci, R. Vari, E. W. Varnes, T. Varol, D. Varouchas, A. Vartapetian, K. E. Varvell, V. I. Vassilakopoulos, F. Vazeille, T. Vazquez Schroeder, F. Veloso, S. Veneziano, A. Ventura, D. Ventura, M. Venturi, N. Venturi, V. Vercesi, M. Verducci, W. Verkerke, J. C. Vermeulen, A. Vest, M. C. Vetterli, I. Vichou, T. Vickey, O. E. Vickey Boeriu, G. H. A. Viehhauser, S. Viel, M. Villa, M. Villaplana Perez, E. Vilucchi, M. G. Vincter, V. B. Vinogradov, J. Virzi, O. Vitells, M. Viti, I. Vivarelli, F. Vives Vaque, S. Vlachos, D. Vladoiu, M. Vlasak, A. Vogel, P. Vokac, G. Volpi, M. Volpi, G. Volpini, H. von der Schmitt, H. von Radziewski, E. von Toerne, V. Vorobel, M. Vos, R. Voss, J. H. Vossebeld, N. Vranjes, M. Vranjes Milosavljevic, V. Vrba, M. Vreeswijk, T. Vu Anh, R. Vuillermet, I. Vukotic, Z. Vykydal, W. Wagner, P. Wagner, S. Wahrmund, J. Wakabayashi, S. Walch, J. Walder, R. Walker, W. Walkowiak, R. Wall, P. Waller, B. Walsh, C. Wang, H. Wang, H. Wang, J. Wang, J. Wang, K. Wang, R. Wang, S. M. Wang, T. Wang, X. Wang, A. Warburton, C. P. Ward, D. R. Wardrope, M. Warsinsky, A. Washbrook, C. Wasicki, I. Watanabe, P. M. Watkins, A. T. Watson, I. J. Watson, M. F. Watson, G. Watts, S. Watts, A. T. Waugh, B. M. Waugh, M. S. Weber, J. S. Webster, A. R. Weidberg, P. Weigell, J. Weingarten, C. Weiser, P. S. Wells, T. Wenaus, D. Wendland, Z. Weng, T. Wengler, S. Wenig, N. Wermes, M. Werner, P. Werner, M. Werth, M. Wessels, J. Wetter, K. Whalen, A. White, M. J. White, R. White, S. White, S. R. Whitehead, D. Whiteson, D. Whittington, D. Wicke, F. J. Wickens, W. Wiedenmann, M. Wielers, P. Wienemann, C. Wiglesworth, L. A. M. Wiik-Fuchs, P. A. Wijeratne, A. Wildauer, M. A. Wildt, I. Wilhelm, H. G. Wilkens, J. Z. Will, E. Williams, H. H. Williams, S. Williams, W. Willis, S. Willocq, J. A. Wilson, A. Wilson, I. Wingerter-Seez, S. Winkelmann, F. Winklmeier, M. Wittgen, T. Wittig, J. Wittkowski, S. J. Wollstadt, M. W. Wolter, H. Wolters, W. C. Wong, G. Wooden, B. K. Wosiek, J. Wotschack, M. J. Woudstra, K. W. Wozniak, K. Wraight, M. Wright, B. Wrona, S. L. Wu, X. Wu, Y. Wu, E. Wulf, B. M. Wynne, S. Xella, M. Xiao, S. Xie, C. Xu, D. Xu, L. Xu, B. Yabsley, S. Yacoob, M. Yamada, H. Yamaguchi, Y. Yamaguchi, A. Yamamoto, K. Yamamoto, S. Yamamoto, T. Yamamura, T. Yamanaka, K. Yamauchi, T. Yamazaki, Y. Yamazaki, Z. Yan, H. Yang, H. Yang, U. K. Yang, Y. Yang, Z. Yang, S. Yanush, L. Yao, Y. Yasu, E. Yatsenko, K. H. Yau Wong, J. Ye, S. Ye, A. L. Yen, E. Yildirim, M. Yilmaz, R. Yoosoofmiya, K. Yorita, R. Yoshida, K. Yoshihara, C. Young, C. J. S. Young, S. Youssef, D. Yu, D. R. Yu, J. Yu, J. Yu, L. Yuan, A. Yurkewicz, B. Zabinski, R. Zaidan, A. M. Zaitsev, S. Zambito, L. Zanello, D. Zanzi, A. Zaytsev, C. Zeitnitz, M. Zeman, A. Zemla, O. Zenin, T. Ženiš, D. Zerwas, G. Zevi della Porta, D. Zhang, H. Zhang, J. Zhang, L. Zhang, X. Zhang, Z. Zhang, Z. Zhao, A. Zhemchugov, J. Zhong, B. Zhou, N. Zhou, Y. Zhou, C. G. Zhu, H. Zhu, J. Zhu, Y. Zhu, X. Zhuang, A. Zibell, D. Zieminska, N. I. Zimin, C. Zimmermann, R. Zimmermann, S. Zimmermann, S. Zimmermann, Z. Zinonos, M. Ziolkowski, R. Zitoun, L. Živković, V. V. Zmouchko, G. Zobernig, A. Zoccoli, M. zur Nedden, V. Zutshi, L. Zwalinski

**Affiliations:** 1 CERN, 1211 Geneva 23, Switzerland; 2 School of Chemistry and Physics, University of Adelaide, Adelaide, Australia; 3 Physics Department, SUNY Albany, Albany, NY United States of America; 4 Department of Physics, University of Alberta, Edmonton, AB Canada; 5 Department of Physics, Ankara University, Ankara, Turkey; 6 Department of Physics, Gazi University, Ankara, Turkey; 7 Division of Physics, TOBB University of Economics and Technology, Ankara, Turkey; 8 Turkish Atomic Energy Authority, Ankara, Turkey; 9 LAPP, CNRS/IN2P3 and Université de Savoie, Annecy-le-Vieux, France; 10 High Energy Physics Division, Argonne National Laboratory, Argonne, IL United States of America; 11 Department of Physics, University of Arizona, Tucson, AZ United States of America; 12 Department of Physics, The University of Texas at Arlington, Arlington, TX United States of America; 13 Physics Department, University of Athens, Athens, Greece; 14 Physics Department, National Technical University of Athens, Zografou, Greece; 15 Institute of Physics, Azerbaijan Academy of Sciences, Baku, Azerbaijan; 16 Institut de Física d’Altes Energies and Departament de Física de la Universitat Autònoma de Barcelona, Barcelona, Spain; 17 Institute of Physics, University of Belgrade, Belgrade, Serbia; 18 Vinca Institute of Nuclear Sciences, University of Belgrade, Belgrade, Serbia; 19 Department for Physics and Technology, University of Bergen, Bergen, Norway; 20 Physics Division, Lawrence Berkeley National Laboratory and University of California, Berkeley, CA United States of America; 21 Department of Physics, Humboldt University, Berlin, Germany; 22 Albert Einstein Center for Fundamental Physics and Laboratory for High Energy Physics, University of Bern, Bern, Switzerland; 23 School of Physics and Astronomy, University of Birmingham, Birmingham, United Kingdom; 24 Department of Physics, Bogazici University, Istanbul, Turkey; 25 Department of Physics, Dogus University, Istanbul, Turkey; 26 Department of Physics Engineering, Gaziantep University, Gaziantep, Turkey; 27 INFN Sezione di Bologna, Bologna, Italy; 28 Dipartimento di Fisica e Astronomia, Università di Bologna, Bologna, Italy; 29 Physikalisches Institut, University of Bonn, Bonn, Germany; 30 Department of Physics, Boston University, Boston, MA United States of America; 31 Department of Physics, Brandeis University, Waltham, MA United States of America; 32 Universidade Federal do Rio De Janeiro COPPE/EE/IF, Rio de Janeiro, Brazil; 33 Federal University of Juiz de Fora (UFJF), Juiz de Fora, Brazil; 34 Federal University of Sao Joao del Rei (UFSJ), Sao Joao del Rei, Brazil; 35 Instituto de Fisica, Universidade de Sao Paulo, Sao Paulo, Brazil; 36 Physics Department, Brookhaven National Laboratory, Upton, NY United States of America; 37 National Institute of Physics and Nuclear Engineering, Bucharest, Romania; 38 Physics Department, National Institute for Research and Development of Isotopic and Molecular Technologies, Cluj Napoca, Romania; 39 University Politehnica Bucharest, Bucharest, Romania; 40 West University in Timisoara, Timisoara, Romania; 41 Departamento de Física, Universidad de Buenos Aires, Buenos Aires, Argentina; 42 Cavendish Laboratory, University of Cambridge, Cambridge, United Kingdom; 43 Department of Physics, Carleton University, Ottawa, ON Canada; 44 CERN, Geneva, Switzerland; 45 Enrico Fermi Institute, University of Chicago, Chicago, IL United States of America; 46 Departamento de Física, Pontificia Universidad Católica de Chile, Santiago, Chile; 47 Departamento de Física, Universidad Técnica Federico Santa María, Valparaíso, Chile; 48 Institute of High Energy Physics, Chinese Academy of Sciences, Beijing, China; 49 Department of Modern Physics, University of Science and Technology of China, Anhui, China; 50 Department of Physics, Nanjing University, Jiangsu, China; 51 School of Physics, Shandong University, Shandong, China; 52 Physics Department, Shanghai Jiao Tong University, Shanghai, China; 53 Laboratoire de Physique Corpusculaire, Clermont Université and Université Blaise Pascal and CNRS/IN2P3, Clermont-Ferrand, France; 54 Nevis Laboratory, Columbia University, Irvington, NY United States of America; 55 Niels Bohr Institute, University of Copenhagen, Kobenhavn, Denmark; 56 INFN Gruppo Collegato di Cosenza, Laboratori Nazionali di Frascati, Rende, Italy; 57 Dipartimento di Fisica, Università della Calabria, Rende, Italy; 58 Faculty of Physics and Applied Computer Science, AGH University of Science and Technology, Krakow, Poland; 59 Marian Smoluchowski Institute of Physics, Jagiellonian University, Krakow, Poland; 60 The Henryk Niewodniczanski Institute of Nuclear Physics, Polish Academy of Sciences, Krakow, Poland; 61 Physics Department, Southern Methodist University, Dallas, TX United States of America; 62 Physics Department, University of Texas at Dallas, Richardson, TX United States of America; 63 DESY, Hamburg and Zeuthen, Germany; 64 Institut für Experimentelle Physik IV, Technische Universität Dortmund, Dortmund, Germany; 65 Institut für Kern- und Teilchenphysik, Technische Universität Dresden, Dresden, Germany; 66 Department of Physics, Duke University, Durham, NC United States of America; 67 SUPA - School of Physics and Astronomy, University of Edinburgh, Edinburgh, United Kingdom; 68 Laboratori Nazionali di Frascati, INFN, Frascati, Italy; 69 Fakultät für Mathematik und Physik, Albert-Ludwigs-Universität, Freiburg, Germany; 70 Section de Physique, Université de Genève, Geneva, Switzerland; 71 INFN Sezione di Genova, Genova, Italy; 72 Dipartimento di Fisica, Università di Genova, Genova, Italy; 73 E. Andronikashvili Institute of Physics, Iv. Javakhishvili Tbilisi State University, Tbilisi, Georgia; 74 High Energy Physics Institute, Tbilisi State University, Tbilisi, Georgia; 75 II Physikalisches Institut, Justus-Liebig-Universität Giessen, Giessen, Germany; 76 SUPA - School of Physics and Astronomy, University of Glasgow, Glasgow, United Kingdom; 77 II Physikalisches Institut, Georg-August-Universität, Göttingen, Germany; 78 Laboratoire de Physique Subatomique et de Cosmologie, Université Joseph Fourier and CNRS/IN2P3 and Institut National Polytechnique de Grenoble, Grenoble, France; 79 Department of Physics, Hampton University, Hampton, VA United States of America; 80 Laboratory for Particle Physics and Cosmology, Harvard University, Cambridge, MA United States of America; 81 Kirchhoff-Institut für Physik, Ruprecht-Karls-Universität Heidelberg, Heidelberg, Germany; 82 Physikalisches Institut, Ruprecht-Karls-Universität Heidelberg, Heidelberg, Germany; 83 ZITI Institut für technische Informatik, Ruprecht-Karls-Universität Heidelberg, Mannheim, Germany; 84 Faculty of Applied Information Science, Hiroshima Institute of Technology, Hiroshima, Japan; 85 Department of Physics, Indiana University, Bloomington, IN United States of America; 86 Institut für Astro- und Teilchenphysik, Leopold-Franzens-Universität, Innsbruck, Austria; 87 University of Iowa, Iowa City, IA United States of America; 88 Department of Physics and Astronomy, Iowa State University, Ames, IA United States of America; 89 Joint Institute for Nuclear Research, JINR Dubna, Dubna, Russia; 90 KEK, High Energy Accelerator Research Organization, Tsukuba, Japan; 91 Graduate School of Science, Kobe University, Kobe, Japan; 92 Faculty of Science, Kyoto University, Kyoto, Japan; 93 Kyoto University of Education, Kyoto, Japan; 94 Department of Physics, Kyushu University, Fukuoka, Japan; 95 Instituto de Física La Plata, Universidad Nacional de La Plata and CONICET, La Plata, Argentina; 96 Physics Department, Lancaster University, Lancaster, United Kingdom; 97 INFN Sezione di Lecce, Lecce, Italy; 98 Dipartimento di Matematica e Fisica, Università del Salento, Lecce, Italy; 99 Oliver Lodge Laboratory, University of Liverpool, Liverpool, United Kingdom; 100 Department of Physics, Jožef Stefan Institute and University of Ljubljana, Ljubljana, Slovenia; 101 School of Physics and Astronomy, Queen Mary University of London, London, United Kingdom; 102 Department of Physics, Royal Holloway University of London, Surrey, United Kingdom; 103 Department of Physics and Astronomy, University College London, London, United Kingdom; 104 Louisiana Tech University, Ruston, LA United States of America; 105 Laboratoire de Physique Nucléaire et de Hautes Energies, UPMC and Université Paris-Diderot and CNRS/IN2P3, Paris, France; 106 Fysiska institutionen, Lunds universitet, Lund, Sweden; 107 Departamento de Fisica Teorica C-15, Universidad Autonoma de Madrid, Madrid, Spain; 108 Institut für Physik, Universität Mainz, Mainz, Germany; 109 School of Physics and Astronomy, University of Manchester, Manchester, United Kingdom; 110 CPPM, Aix-Marseille Université and CNRS/IN2P3, Marseille, France; 111 Department of Physics, University of Massachusetts, Amherst, MA United States of America; 112 Department of Physics, McGill University, Montreal, QC Canada; 113 School of Physics, University of Melbourne, Victoria, Australia; 114 Department of Physics, The University of Michigan, Ann Arbor, MI United States of America; 115 Department of Physics and Astronomy, Michigan State University, East Lansing, MI United States of America; 116 INFN Sezione di Milano, Milano, Italy; 117 Dipartimento di Fisica, Università di Milano, Milano, Italy; 118 B.I. Stepanov Institute of Physics, National Academy of Sciences of Belarus, Minsk, Republic of Belarus; 119 National Scientific and Educational Centre for Particle and High Energy Physics, Minsk, Republic of Belarus; 120 Department of Physics, Massachusetts Institute of Technology, Cambridge, MA United States of America; 121 Group of Particle Physics, University of Montreal, Montreal, QC Canada; 122 P.N. Lebedev Institute of Physics, Academy of Sciences, Moscow, Russia; 123 Institute for Theoretical and Experimental Physics (ITEP), Moscow, Russia; 124 Moscow Engineering and Physics Institute (MEPhI), Moscow, Russia; 125 D.V. Skobeltsyn Institute of Nuclear Physics, M.V.Lomonosov Moscow State University, Moscow, Russia; 126 Fakultät für Physik, Ludwig-Maximilians-Universität München, München, Germany; 127 Max-Planck-Institut für Physik (Werner-Heisenberg-Institut), München, Germany; 128 Nagasaki Institute of Applied Science, Nagasaki, Japan; 129 Graduate School of Science and Kobayashi-Maskawa Institute, Nagoya University, Nagoya, Japan; 130 INFN Sezione di Napoli, Napoli, Italy; 131 Dipartimento di Scienze Fisiche, Università di Napoli, Napoli, Italy; 132 Department of Physics and Astronomy, University of New Mexico, Albuquerque, NM United States of America; 133 Institute for Mathematics, Astrophysics and Particle Physics, Radboud University Nijmegen/Nikhef, Nijmegen, Netherlands; 134 Nikhef National Institute for Subatomic Physics and University of Amsterdam, Amsterdam, Netherlands; 135 Department of Physics, Northern Illinois University, DeKalb, IL United States of America; 136 Budker Institute of Nuclear Physics, SB RAS, Novosibirsk, Russia; 137 Department of Physics, New York University, New York, NY United States of America; 138 Ohio State University, Columbus, OH United States of America; 139 Faculty of Science, Okayama University, Okayama, Japan; 140 Homer L. Dodge Department of Physics and Astronomy, University of Oklahoma, Norman, OK United States of America; 141 Department of Physics, Oklahoma State University, Stillwater, OK United States of America; 142 RCPTM, Palacký University, Olomouc, Czech Republic; 143 Center for High Energy Physics, University of Oregon, Eugene, OR United States of America; 144 LAL, Université Paris-Sud and CNRS/IN2P3, Orsay, France; 145 Graduate School of Science, Osaka University, Osaka, Japan; 146 Department of Physics, University of Oslo, Oslo, Norway; 147 Department of Physics, Oxford University, Oxford, United Kingdom; 148 INFN Sezione di Pavia, Pavia, Italy; 149 Dipartimento di Fisica, Università di Pavia, Pavia, Italy; 150 Department of Physics, University of Pennsylvania, Philadelphia, PA United States of America; 151 Petersburg Nuclear Physics Institute, Gatchina, Russia; 152 INFN Sezione di Pisa, Pisa, Italy; 153 Dipartimento di Fisica E. Fermi, Università di Pisa, Pisa, Italy; 154 Department of Physics and Astronomy, University of Pittsburgh, Pittsburgh, PA United States of America; 155 Laboratorio de Instrumentacao e Fisica Experimental de Particulas - LIP, Lisboa, Portugal; 156 Departamento de Fisica Teorica y del Cosmos and CAFPE, Universidad de Granada, Granada, Spain; 157 Institute of Physics, Academy of Sciences of the Czech Republic, Praha, Czech Republic; 158 Czech Technical University in Prague, Praha, Czech Republic; 159 Faculty of Mathematics and Physics, Charles University in Prague, Praha, Czech Republic; 160 State Research Center Institute for High Energy Physics, Protvino, Russia; 161 Particle Physics Department, Rutherford Appleton Laboratory, Didcot, United Kingdom; 162 Physics Department, University of Regina, Regina, SK Canada; 163 Ritsumeikan University, Kusatsu, Shiga Japan; 164 INFN Sezione di Roma I, Roma, Italy; 165 Dipartimento di Fisica, Università La Sapienza, Roma, Italy; 166 INFN Sezione di Roma Tor Vergata, Roma, Italy; 167 Dipartimento di Fisica, Università di Roma Tor Vergata, Roma, Italy; 168 INFN Sezione di Roma Tre, Roma, Italy; 169 Dipartimento di Matematica e Fisica, Università Roma Tre, Roma, Italy; 170 Faculté des Sciences Ain Chock, Réseau Universitaire de Physique des Hautes Energies - Université Hassan II, Casablanca, Morocco; 171 Centre National de l’Energie des Sciences Techniques Nucleaires, Rabat, Morocco; 172 Faculté des Sciences Semlalia, Université Cadi Ayyad, LPHEA-Marrakech, Morocco; 173 Faculté des Sciences, Université Mohamed Premier and LPTPM, Oujda, Morocco; 174 Faculté des sciences, Université Mohammed V-Agdal, Rabat, Morocco; 175 DSM/IRFU (Institut de Recherches sur les Lois Fondamentales de l’Univers), CEA Saclay (Commissariat à l’Energie Atomique et aux Energies Alternatives), Gif-sur-Yvette, France; 176 Santa Cruz Institute for Particle Physics, University of California Santa Cruz, Santa Cruz, CA United States of America; 177 Department of Physics, University of Washington, Seattle, WA United States of America; 178 Department of Physics and Astronomy, University of Sheffield, Sheffield, United Kingdom; 179 Department of Physics, Shinshu University, Nagano, Japan; 180 Fachbereich Physik, Universität Siegen, Siegen, Germany; 181 Department of Physics, Simon Fraser University, Burnaby, BC Canada; 182 SLAC National Accelerator Laboratory, Stanford, CA United States of America; 183 Faculty of Mathematics, Physics & Informatics, Comenius University, Bratislava, Slovak Republic; 184 Department of Subnuclear Physics, Institute of Experimental Physics of the Slovak Academy of Sciences, Kosice, Slovak Republic; 185 Department of Physics, University of Cape Town, Cape Town, South Africa; 186 Department of Physics, University of Johannesburg, Johannesburg, South Africa; 187 School of Physics, University of the Witwatersrand, Johannesburg, South Africa; 188 Department of Physics, Stockholm University, Stockholm, Sweden; 189 The Oskar Klein Centre, Stockholm, Sweden; 190 Physics Department, Royal Institute of Technology, Stockholm, Sweden; 191 Departments of Physics & Astronomy and Chemistry, Stony Brook University, Stony Brook, NY United States of America; 192 Department of Physics and Astronomy, University of Sussex, Brighton, United Kingdom; 193 School of Physics, University of Sydney, Sydney, Australia; 194 Institute of Physics, Academia Sinica, Taipei, Taiwan; 195 Department of Physics, Technion: Israel Institute of Technology, Haifa, Israel; 196 Raymond and Beverly Sackler School of Physics and Astronomy, Tel Aviv University, Tel Aviv, Israel; 197 Department of Physics, Aristotle University of Thessaloniki, Thessaloniki, Greece; 198 International Center for Elementary Particle Physics and Department of Physics, The University of Tokyo, Tokyo, Japan; 199 Graduate School of Science and Technology, Tokyo Metropolitan University, Tokyo, Japan; 200 Department of Physics, Tokyo Institute of Technology, Tokyo, Japan; 201 Department of Physics, University of Toronto, Toronto, ON Canada; 202 TRIUMF, Vancouver, BC Canada; 203 Department of Physics and Astronomy, York University, Toronto, ON Canada; 204 Faculty of Pure and Applied Sciences, University of Tsukuba, Tsukuba, Japan; 205 Department of Physics and Astronomy, Tufts University, Medford, MA United States of America; 206 Centro de Investigaciones, Universidad Antonio Narino, Bogota, Colombia; 207 Department of Physics and Astronomy, University of California Irvine, Irvine, CA United States of America; 208 INFN Gruppo Collegato di Udine, Sezione di Trieste, Udine, Italy; 209 ICTP, Trieste, Italy; 210 Dipartimento di Chimica, Fisica e Ambiente, Università di Udine, Udine, Italy; 211 Department of Physics, University of Illinois, Urbana, IL United States of America; 212 Department of Physics and Astronomy, University of Uppsala, Uppsala, Sweden; 213 Instituto de Física Corpuscular (IFIC) and Departamento de Física Atómica, Molecular y Nuclear and Departamento de Ingeniería Electrónica and Instituto de Microelectrónica de Barcelona (IMB-CNM), University of Valencia and CSIC, Valencia, Spain; 214 Department of Physics, University of British Columbia, Vancouver, BC Canada; 215 Department of Physics and Astronomy, University of Victoria, Victoria, BC Canada; 216 Department of Physics, University of Warwick, Coventry, United Kingdom; 217 Waseda University, Tokyo, Japan; 218 Department of Particle Physics, The Weizmann Institute of Science, Rehovot, Israel; 219 Department of Physics, University of Wisconsin, Madison, WI United States of America; 220 Fakultät für Physik und Astronomie, Julius-Maximilians-Universität, Würzburg, Germany; 221 Fachbereich C Physik, Bergische Universität Wuppertal, Wuppertal, Germany; 222 Department of Physics, Yale University, New Haven, CT United States of America; 223 Yerevan Physics Institute, Yerevan, Armenia; 224 Centre de Calcul de l’Institut National de Physique Nucléaire et de Physique des Particules (IN2P3), Villeurbanne, France

## Abstract

A measurement of jet shapes in top-quark pair events using 1.8 fb^−1^ of $\sqrt{s} = 7 \ \mbox{TeV}$ *pp* collision data recorded by the ATLAS detector at the LHC is presented. Samples of top-quark pair events are selected in both the single-lepton and dilepton final states. The differential and integrated shapes of the jets initiated by bottom-quarks from the top-quark decays are compared with those of the jets originated by light-quarks from the hadronic *W*-boson decays $W\rightarrow q\bar{q}'$ in the single-lepton channel. The light-quark jets are found to have a narrower distribution of the momentum flow inside the jet area than *b*-quark jets.

## Introduction

Hadronic jets are observed in large momentum-transfer interactions. They are theoretically interpreted to arise when partons—quarks (*q*) and gluons (*g*)—are emitted in collision events of subatomic particles. Partons then evolve into hadronic jets in a two-step process. The first can be described by perturbation theory and gives rise to a parton shower, the second is non-perturbative and is responsible for the hadronisation. The internal structure of a jet is expected to depend primarily on the type of parton it originated from, with some residual dependence on the quark production and fragmentation process. For instance, due to the different colour factors in *ggg* and *qqg* vertices, gluons lead to more parton radiation and therefore gluon-initiated jets are expected to be broader than quark-initiated jets.

For jets defined using cone or *k*_*t*_ algorithms [1, 2], jet shapes, i.e. the normalised transverse momentum flow as a function of the distance to the jet axis [3], have been traditionally used as a means of understanding the evolution of partons into hadrons in *e* ^+^ *e* ^−^, *ep* and hadron colliders [4–11]. It is experimentally observed that jets in *e* ^+^ *e* ^−^ and *ep* are narrower than those observed in $p\bar{p}$ and *pp* collisions and this is interpreted as a result of the different admixtures of quark and gluon jets present in these different types of interactions [12]. Furthermore, at high momentum transfer, where fragmentation effects are less relevant, jet shapes have been found to be in qualitative agreement with next-to-leading-order (NLO) QCD predictions and in quantitative agreement with those including leading logarithm corrections [13]. Jet shapes have also been proposed as a tool for studies of substructure or in searches for new phenomena in final states with highly boosted particles [14–17].

Due to the mass of the *b*-quark, jets originating from a *b*-quark (hereafter called *b*-jets) are expected to be broader than light-quark jets, including charm jets, hereafter called light jets. This expectation is supported by observations by the CDF collaboration in Ref. [18], where a comparison is presented between jet shapes in a *b*-jet enriched sample with a purity of roughly 25 % and an inclusive sample where no distinction is made between the flavours.

This paper presents the first measurement of *b*-jet shapes in top pair events. The $t\bar{t}$ final states are a source of *b*-jets, as the top quark decays almost exclusively via *t*→*Wb*. While the dilepton channel, where both *W* bosons decay to leptons, is a very pure source of *b*-jets, the single-lepton channel contains *b*-jets and light jets, the latter originating from the dominant $W^{+} \rightarrow u\bar{d}, c\bar{s}$ decays and their charge conjugates. A comparison of the light- and *b*-jet shapes measured in the $t\bar {t}$ decays improves the CDF measurement discussed above, as the jet purity achieved using $t\bar{t}$ events is much higher. In addition, these measurements could be used to improve the modelling of jets in $t\bar{t}$ production Monte Carlo (MC) models in a new kinematic regime.

This paper is organised as follows. In Sect. 2 the ATLAS detector is described, while Sect. 3 is dedicated to the MC samples used in the analysis. In Sects. 4 and 5, the physics object and event selection for both the dilepton and single-lepton $t\bar{t}$ samples is presented. Section 6 is devoted to the description of both the *b*-jet and light-jet samples obtained in the single-lepton final state. The differential and the integrated shape distributions of these jets are derived in Sect. 7. In Sect. 8 the results on the average values of the jet shape variables at the detector level are presented, including those for the *b*-jets in the dilepton channel. Results corrected for detector effects are presented in Sect. 9. In Sect. 10 the systematic uncertainties are discussed, and Sect. 11 contains a discussion of the results. Finally, Sect. 12 includes the summary and conclusions.

## The ATLAS detector

The ATLAS detector [19] is a multi-purpose particle physics detector with a forward-backward symmetric cylindrical geometry^1^ and a solid angle coverage of almost 4*π*.

The inner tracking system covers the pseudorapidity range |*η*|<2.5, and consists of a silicon pixel detector, a silicon microstrip detector, and, for |*η*|<2.0, a transition radiation tracker. The inner detector (ID) is surrounded by a thin superconducting solenoid providing a 2 T magnetic field along the beam direction. A high-granularity liquid-argon sampling electromagnetic calorimeter covers the region |*η*|<3.2. An iron/scintillator tile hadronic calorimeter provides coverage in the range |*η*|<1.7. The endcap and forward regions, spanning 1.5<|*η*|<4.9, are instrumented with liquid-argon calorimeters for electromagnetic and hadronic measurements. The muon spectrometer surrounds the calorimeters. It consists of three large air-core superconducting toroid systems and separate trigger and high-precision tracking chambers providing accurate muon tracking for |*η*|<2.7.

The trigger system [20] has three consecutive levels: level 1 (L1), level 2 (L2) and the event filter (EF). The L1 triggers are hardware-based and use coarse detector information to identify regions of interest, whereas the L2 triggers are based on fast software-based online data reconstruction algorithms. Finally, the EF triggers use offline data reconstruction algorithms. For this analysis, the relevant triggers select events with at least one electron or muon.

## Monte Carlo samples

Monte Carlo generators are used in which $t\bar{t}$ production is implemented with matrix elements calculated up to NLO accuracy. The generated events are then passed through a detailed Geant4 simulation [21, 22] of the ATLAS detector. The baseline MC samples used here are produced with the MC@NLO [23] or Powheg [24] generators for the matrix element calculation; the parton shower and hadronisation processes are implemented with Herwig [25] using the cluster hadronisation model [26] and CTEQ6.6 [27] parton distribution functions (PDFs). Multi-parton interactions are simulated using Jimmy [28] with the AUET1 tune [29]. This MC generator package has been used for the description of the $t\bar{t}$ final states for ATLAS measurements of the cross section [30, 31] and studies of the kinematics [32].

Additional MC samples are used to check the hadronisation model dependence of the jet shapes. They are based on Powheg+Pythia [24, 33], with the MRST2007LO* PDFs [34]. The AcerMC generator [35] interfaced to Pythia with the Perugia 2010 tune [36] for parton showering and hadronisation is also used for comparison. Here the parton showers are ordered by transverse momentum and the hadronisation proceeds through the Lund string fragmentation scheme [37]. The underlying event and other soft effects are simulated by Pythia with the AMBT1 tune [38]. Comparisons of different event generators show that jet shapes in top-quark decays show little sensitivity to initial-state radiation effects, different PDF choices or underlying-event effects. They are more sensitive to details of the parton shower and the fragmentation scheme.

Samples of events including *W* and *Z* bosons produced in association with light- and heavy-flavour jets are generated using the Alpgen [39] generator with the CTEQ6L PDFs [40], and interfaced with Herwig and Jimmy. The same generator is used for the diboson backgrounds, *WW*, *WZ* and *ZZ*, while MC@NLO is used for the simulation of the single-top backgrounds, including the *t*- and *s*-channels as well as the *Wt*-channel.

The MC-simulated samples are normalised to the corresponding cross sections. The $t\bar{t}$ signal is normalised to the cross section calculated at approximate next-to-next-to-leading order (NNLO) using the Hathor package [41], while for the single-top production cross section, the calculations in Refs. [42–44] are used. The *W*+jets and *Z*+jets cross sections are taken from Alpgen [39] with additional NNLO *K*-factors as given in Ref. [45].

The simulated events are weighted such that the distribution of the number of interactions per bunch crossing in the simulated samples matches that of the data. Finally, additional correction factors are applied to take into account the different object efficiencies in data and simulation. The scale factors used for these corrections typically differ from unity by 1 % for electrons and muons, and by a few percent for *b*-tagging.

## Physics object selection

Electron candidates are reconstructed from energy deposits in the calorimeter that are associated with tracks reconstructed in the ID. The candidates must pass a tight selection [46], which uses calorimeter and tracking variables as well as transition radiation for |*η*|<2.0, and are required to have transverse momentum *p*_T_>25 GeV and |*η*|<2.47. Electrons in the transition region between the barrel and endcap calorimeters, 1.37<|*η*|<1.52, are not considered.

Muon candidates are reconstructed by searching for track segments in different layers of the muon spectrometer. These segments are combined and matched with tracks found in the ID. The candidates are refitted using the complete track information from both detector systems and are required to have a good fit and to satisfy *p*_T_>20 GeV and |*η*|<2.5.

Electron and muon candidates are required to be isolated to reduce backgrounds arising from jets and to suppress the selection of leptons from heavy-flavour semileptonic decays. For electron candidates, the transverse energy deposited in the calorimeter and which is not associated with the electron itself ($E^{\mathrm{iso}}_{\mathrm{T}}$) is summed in a cone in *η*–*ϕ* space of radius^2^ *ΔR*=0.2 around the electron. The $E^{\mathrm{iso}}_{\mathrm{T}}$ value is required to be less than 3.5 GeV. For muon candidates, both the corresponding calorimeter isolation $E^{\mathrm{iso}}_{\mathrm{T}}$ and the analogous track isolation transverse momentum ($p^{\mathrm{iso}}_{\mathrm{T}}$) must be less than 4 GeV in a cone of *ΔR*=0.3. The track isolation is calculated from the scalar sum of the transverse momenta of tracks with *p*_T_>1 GeV, excluding the muon.

Muon candidates arising from cosmic rays are rejected by removing candidate pairs that are back-to-back in the transverse plane and that have transverse impact parameter relative to the beam axis |*d*_0_|>0.5 mm.

Jets are reconstructed with the anti-*k*_*t*_ algorithm [47, 48] with radius parameter *R*=0.4. This choice for the radius has been used in measurements of the top-quark mass [49] and also in multi-jet cross-section measurements [50]. The inputs to the jet algorithm are topological clusters of calorimeter cells. These clusters are seeded by calorimeter cells with energy |*E*_cell_|>4*σ*, where *σ* is the cell-by-cell RMS of the noise (electronics plus pileup). Neighbouring cells are added if |*E*_cell_|>2*σ* and clusters are formed through an iterative procedure [51]. In a final step, all remaining neighbouring cells are added to the cluster.

The baseline calibration for these clusters calculates their energy using the electromagnetic energy scale [54]. This is established using test-beam measurements for electrons and muons in the electromagnetic and hadronic calorimeters [51–53]. Effects due to the differing response to electromagnetic and hadronic showers, energy losses in the dead material, shower leakage, as well as inefficiencies in energy clustering and jet reconstruction are also taken into account. This is done by matching calorimeter jets with MC particle jets in bins of *η* and *E*, and supplemented by in situ calibration methods such as jet momentum imbalance in *Z*/*γ* ^∗^+1 jet events. This is called the Jet Energy Scale (JES) calibration, thoroughly discussed in Ref. [54]. The JES uncertainty contains an extra term for *b*-quark jets, as the jet response is different for *b*-jets and light jets because they have different particle composition. References [50] and [55] contain more details on the JES and a discussion of its uncertainties.

Jets that overlap with a selected electron are removed if they are closer than *ΔR*=0.2, while if a jet is closer than *ΔR*=0.4 to a muon, the muon is removed.

The primary vertex is defined as the *pp* interaction vertex with the largest $\sum_{i} p_{\mathrm{T}i}^{2}$, where the sum runs over the tracks with *p*_T_>150 MeV associated with the vertex.

Jets are identified as candidates for having originated from a *b*-quark (*b*-tagged) by an algorithm based on a neural-network approach, as discussed in Sect. 6.

The reconstruction of the direction and magnitude ($E_{\mathrm {T}}^{\mathrm{miss}}$) of the missing transverse momentum is described in Ref. [56] and begins with the vector sum of the transverse momenta of all jets with *p*_T_>20 GeV and |*η*|<4.5. The transverse momenta of electron candidates are added. The contributions from all muon candidates and from all calorimeter clusters not belonging to a reconstructed object are also included.

## Event selection

**Fig. 1:**
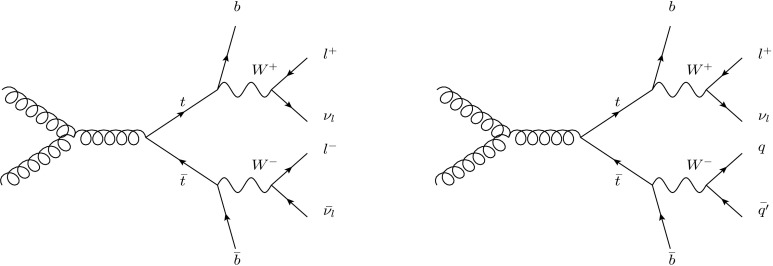
Example LO Feynman diagrams for $gg\rightarrow t\bar{t}$ in the dilepton (*left*) and single-lepton (*right*) decay modes

Two samples of events are selected: a dilepton sample, where both *W* bosons decay to leptons (*e*, *μ*, including leptonic *τ* decays), and a single-lepton sample, where one *W* boson decays to leptons and the other to a $q\bar{q}'$ pair, giving rise to two more jets (see Fig. 1). The selection criteria follow those in Ref. [30] for the single-lepton sample and Ref. [31] for the dilepton sample. Events are triggered by inclusive high-*p*_T_ electron or muon EF triggers. The trigger thresholds are 18 GeV for muons and 20 GeV for electrons. The dataset used for the analysis corresponds to the first half of the data collected in 2011, with a centre-of-mass energy $\sqrt{s} = 7 \ \mbox{TeV}$ and an integrated luminosity of 1.8 fb^−1^. This data-taking period is characterised by an instantaneous luminosity smaller than 1.5×10^33^ cm^−2^ s^−1^, for which the mean number of interactions per bunch crossing is less than six. To reject the non-collision background, the primary vertex is required to have at least four tracks, each with *p*_T_>150 MeV, associated with it. Pile-up effects are therefore small and have been taken into account as a systematic uncertainty.

### Dilepton sample

In the dilepton sample, events are required to have two charged leptons and $E_{\mathrm{T}}^{\mathrm{miss}}$ from the leptonic *W*-boson decays to a neutrino and an electron or muon. The offline lepton selection requires two isolated leptons (*e* or *μ*) with opposite charge and with transverse momenta *p*_T_(*e*)>25 GeV, where *p*_T_(*e*)=*E*_cluster_sin(*θ*_track_), *E*_cluster_ being the cluster energy and *θ*_track_ the track polar angle, and *p*_T_(*μ*)>20 GeV. At least one of the selected leptons has to match the corresponding trigger object.

Events are further filtered by requiring at least two jets with *p*_T_>25 GeV and |*η*|<2.5 in the event. In addition, at least one of the selected jets has to be tagged as a *b*-jet, as discussed in the next section. The whole event is rejected if a jet is identified as an out-of-time signal or as noise in the calorimeter.

The missing transverse momentum requirement is $E_{\mathrm {T}}^{\mathrm{miss}} > 60 \ \mbox{GeV}$ for the *ee* and *μμ* channels. For the *eμ* channel, *H*_T_ is required to be greater than 130 GeV, where *H*_T_ is the scalar sum of the *p*_T_ of all muons, electrons and jets. To reject the Drell–Yan lepton pair background in the *ee* and *μμ* channels, the lepton pair is required to have an invariant mass *m*_*ℓℓ*_ greater than 15 GeV and to lie outside of a *Z*-boson mass window, rejecting all events where the two-lepton invariant mass satisfies |*m*_*ℓℓ*_−*m*_*Z*_|<10 GeV.

The selected sample consists of 95 % $t\bar{t}$ events, but also backgrounds from the final states *W*+jets and *Z*+jets, where the gauge bosons decay to leptons. All backgrounds, with the exception of multi-jet production, have been estimated using MC samples. The multi-jet background has been estimated using the jet–electron method [60]. This method relies on the identification of jets which, due to their high electromagnetic energy fraction, can fake electron candidates. The jet–electron method is applied with some modifications to the muon channel as well. The normalisation is estimated using a binned likelihood fit to the $E_{\mathrm{T}}^{\mathrm{miss}} $ distribution. The results are summarised in Table 1.

**Table 1:** The expected composition of the dilepton sample. Fractions are relative to the total number of expected events. ‘Other EW’ corresponds to the *W*+jets and diboson (*WW*, *WZ* and *ZZ*) contributions

Process	Expected events	Fraction
$t\bar{t}$	2100±110	94.9 %
*Z*+jets (*Z*→*ℓ* ^+^ *ℓ* ^−^)	14±1	0.6 %
Other EW (*W*, diboson)	4±2	0.2 %
Single top	95±2	4.3 %
Multi-jet	$0^{+2}_{-0}$	0.0 %
**Total Expected**	2210±110	
**Total Observed**	2067	

### Single-lepton sample

In this case, the event is required to have exactly one isolated lepton with *p*_T_>25 GeV for electrons and *p*_T_>20 GeV for muons. To account for the neutrino in the leptonic *W* decay, $E_{\mathrm {T}}^{\mathrm{miss}}$ is required to be greater than 35 GeV in the electron channel and greater than 20 GeV in the muon channel. The $E_{\mathrm {T}}^{\mathrm{miss}}$ resolution is below 10 GeV [56]. Furthermore, the transverse mass^3^ (*m*_T_) is required to be greater than 25 GeV in the *e*-channel and to satisfy the condition $E_{\mathrm{T}}^{\mathrm{miss}}+m_{\mathrm{T}} > 60 \ \mbox{GeV}$ in the *μ*-channel.

The jet selection requires at least four jets (*p*_T_>25 GeV and |*η*|<2.5) in the final state, and at least one of them has to be tagged as a *b*-jet. The fraction of $t\bar{t}$ events in the sample is 77 %; the main background contributions for the single-lepton channel have been studied as in the previous case, and are summarised in Table 2. As in the dileptonic case, the multi-jet background has been estimated using the jet–electron method.

**Table 2:** The expected composition of the single-lepton sample. Fractions are relative to the total number of expected events. In this case ‘Other EW’ includes *Z*+jets and diboson processes

Process	Expected events	Fraction
$t\bar{t}$	14000±700	77.4 %
*W*+jets (*W*→*ℓν*)	2310±280	12.8 %
Other EW (*Z*, diboson)	198±18	1.1 %
Single top	668±14	3.7 %
Multi-jet	900±450	5.0 %
**Total Expected**	18000±900	
**Total Observed**	17019	

## Jet sample definition

Jets reconstructed in the single-lepton and dilepton samples are now subdivided into *b*-jet and light-jet samples. In order to avoid contributions from non-primary collisions, it is required that the jet vertex fraction (JVF) be greater than 0.75. After summing the scalar *p*_T_ of all tracks in a jet, the JVF is defined as the fraction of the total scalar *p*_T_ that belongs to tracks originating from the primary vertex. This makes the average jet multiplicity independent of the number of *pp* interaction vertices. This selection is not applied to jets with no associated tracks. Also, to reduce the impact of pileup on the jets, the *p*_T_ threshold has been raised to 30 GeV.

Jets whose axes are closer than *ΔR*=0.8, which is twice the jet radius, to some other jet in the event are not considered. This is done to avoid possible overlaps between the jet cones, which would bias the shape measurement. These configurations are typical in boosted *W* bosons, leading to light jets which are not well separated. The resulting *ΔR* distributions for any pair of *b*-jets or light jets are approximately constant between 0.8 and *π* and exhibit an exponential fall-off between *π* and the endpoint of the distribution.

### *b*-jet samples

To select *b*-jets, a neural-network algorithm, which relies on the reconstruction of secondary vertices and impact parameter information in the three spatial dimensions, is used. The reconstruction of the secondary decay vertices makes use of an iterative Kalman-filter algorithm [61] which relies on the hypothesis that the *b*→*c*→*X* decay chains lie in a straight line originally taken to be parallel to the jet axis. The working point of the algorithm is chosen to maximise the purity of the sample. It corresponds to a *b*-tagging efficiency of 57 % for jets originating from *b*-quarks in simulated $t\bar{t}$ events, and a *u*,*d*,*s*-quark jet rejection factor of about 400, as well as a *c*-jet rejection factor of about 10 [62, 63]. The resulting number of *b*-jets selected in the dilepton (single-lepton) sample is 2279 (16735). A second working point with a *b*-tagging efficiency of 70 % is also used in order to evaluate the dependence of the measured jet shapes on *b*-tagging.

**Fig. 2:**
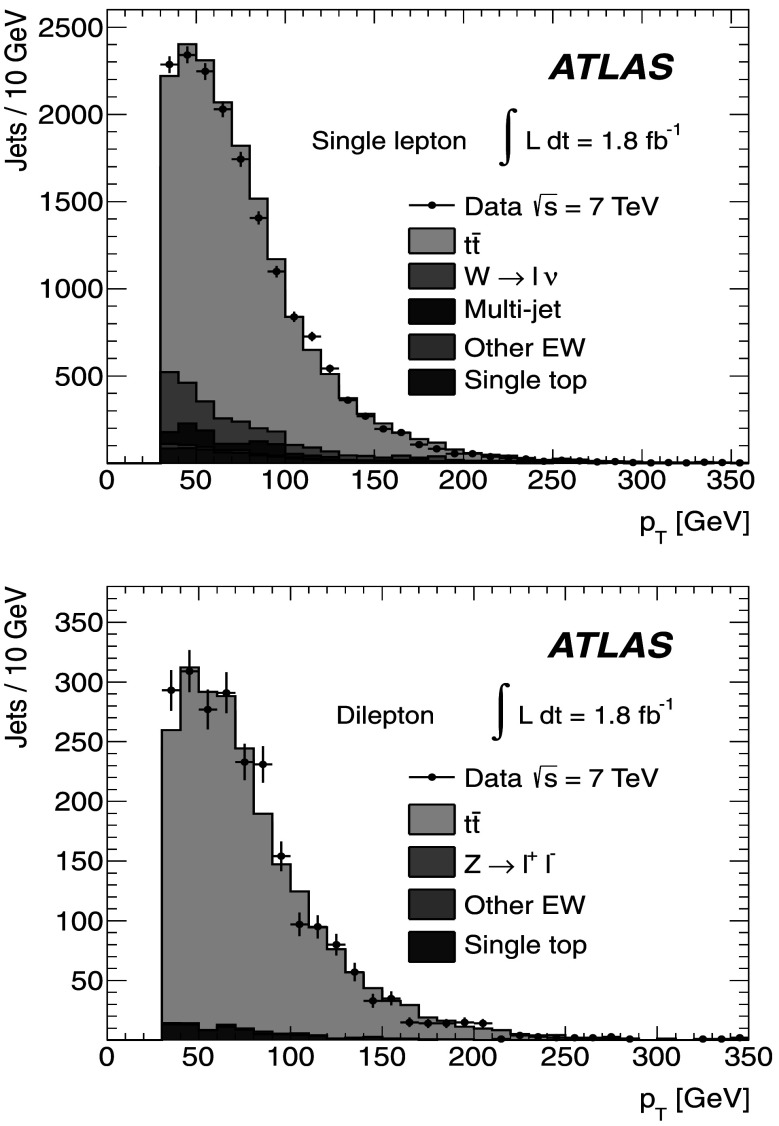
The *p*_T_ distributions for *b*-tagged jets in the single-lepton (*top*) and dilepton (*bottom*) samples along with the sample composition expectations

Figure 2 shows the *b*-tagged jet transverse momentum distributions for the single-lepton and dilepton channels. The *p*_T_ distributions for the *b*-jets in both the dilepton and single-lepton samples show a similar behaviour, since they come mainly from top-quark decays. This is well described by the MC expectations from the MC@NLO generator coupled to Herwig. In the dilepton sample the signal-to-background ratio is found to be greater than in the single-lepton sample, as it is quantitatively shown in Tables 1 and 2.

### Light-quark jet sample

**Fig. 3:**
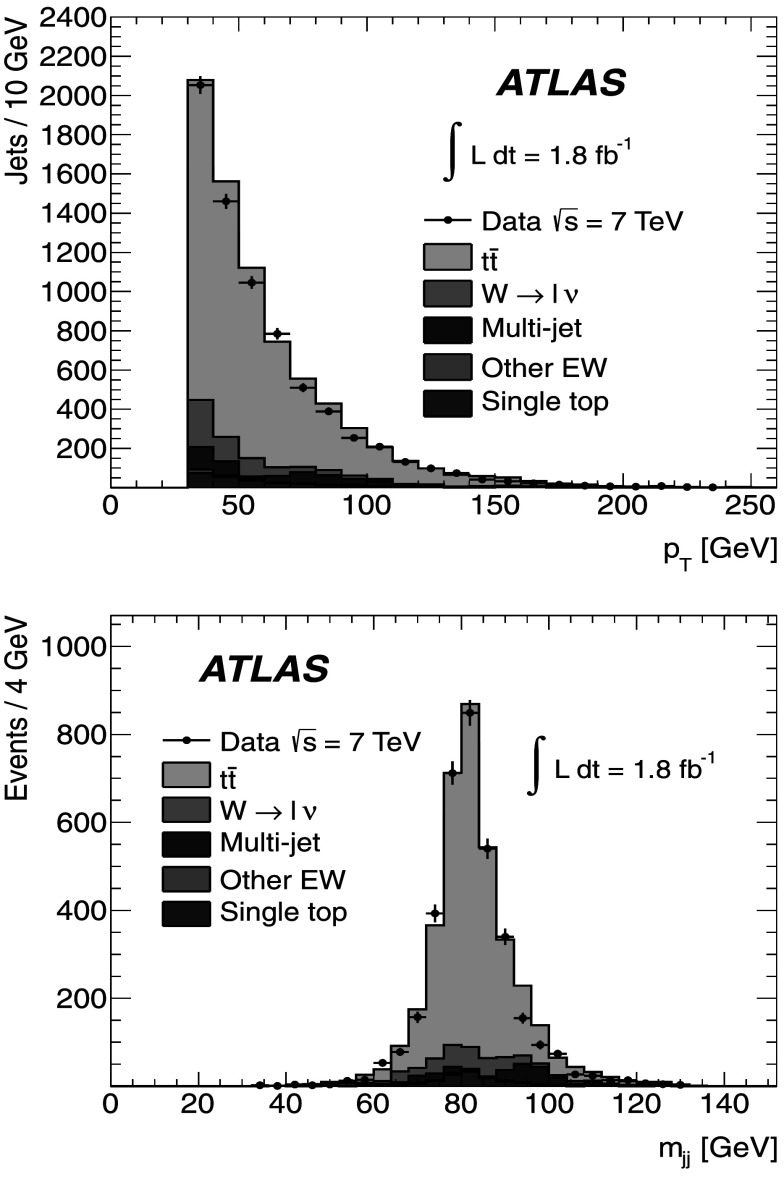
The distribution of light-jet *p*_T_ (*top*) and of the invariant mass of light-jet pairs (*bottom*) along with the sample composition expectations. The latter shows a peak at the *W* mass, whose width is determined by the dijet mass resolution

The hadronic decays $W\rightarrow q\bar{q}'$ are a clean source of light-quark jets, as gluons and *b*-jets are highly suppressed; the former because gluons would originate in radiative corrections of order $\mathcal{O}(\alpha_{s})$, and the latter because of the smallness of the CKM matrix elements |*V*_*ub*_| and |*V*_*cb*_|. To select the light-jet sample, the jet pair in the event which has the invariant mass closest to the *W*-boson mass is selected. Both jets are also required to be non-tagged by the *b*-tagging algorithm. The number of jets satisfying these criteria is 7158. Figure 3 shows the transverse momentum distribution of these jets together with the invariant mass of the dijet system. As expected, the *p*_T_ distribution of the light jets from *W*-boson decays exhibits a stronger fall-off than that for the *b*-jets. This dependence is again well described by the MC simulations in the jet *p*_T_ region used in this analysis. Agreement between the invariant mass distributions for observed and simulated events is good, in particular in the region close to the *W*-boson mass.

### Jet purities

To estimate the actual number of *b*-jets and light jets in each of the samples, the MC simulation is used by analysing the information at generator level. For *b*-jets, a matching to a *b*-hadron is performed within a radius *ΔR*=0.3. For light jets, the jet is required not to have a *b*-hadron within *ΔR*=0.3 of the jet axis. Additionally, to distinguish light quarks and *c*-quarks from gluons, the MC parton with highest *p*_T_ within the cone of the reconstructed jet is required to be a (*u*,*d*,*c* or *s*)-quark. The purity *p* is then defined as

1 $$\begin{aligned} p = \sum_{k}\alpha_{k}p_{k}; \quad p_{k} = 1-\frac{N_{\mathrm {f}}^{(k)}}{N_{\mathrm{T}}^{(k)}} \end{aligned}$$

where *α*_*k*_ is the fraction of events in the *k*-th MC sample (signal or background), given in Tables 1 and 2 and $N_{\mathrm{f}}^{(k)}$, $N_{\mathrm{T}}^{(k)}$ are the number of fakes (jets not assigned to the correct flavour, e.g. charm jets in the *b*-jet sample), and the total number of jets in a given sample, respectively. The purity in the multi-jet background is determined using Pythia MC samples.

In the single-lepton channel, the resulting purity of the *b*-jet sample is $p^{(\mathrm{s})}_{b}=(88.5 \pm5.7)~\%$, while the purity of the light-jet sample is found to be $p^{(\mathrm{s})}_{\mathrm{l}}= (66.2 \pm4.1)~\%$, as shown in Table 3. The uncertainty on the purity arises from the uncertainties on the signal and background fractions in each sample. The charm content in the light-jet sample is found to be 16 %, with the remaining 50 % ascribed to *u*,*d* and *s*.

**Table 3:** Purity estimation for *b*-jets and light jets in the single-lepton channel. The uncertainty on the purity arises from the uncertainties in the signal and background fractions

Process	*α*_*k*_	*p*_*k*_(*b*)	*p*_*k*_ (light)
$t\bar{t}$	0.774	0.961	0.725
*W*→*ℓν*	0.128	0.430	0.360
Multi-jet	0.050	0.887	0.485
Other EW (*Z*, diboson)	0.011	0.611	0.342
Single top	0.037	0.958	0.716
**Weighted total**	–	(88.5±5.7) %	(66.2±4.1) %

MC studies indicate that the contamination of the *b*-jet sample is dominated by charm-jet fakes and that the gluon contamination is about 0.7 %. For the light-jet sample, the fraction of gluon fakes amounts to 19 %, while the *b*-jet fakes correspond to 15 %.

In the dilepton channel, a similar calculation yields the purity of the *b*-jet sample to be $p^{(\mathrm{d})}_{b} = (99.3^{+0.7}_{-6.5})~\%$ as shown in Table 4. Thus, the *b*-jet sample purity achieved using $t\bar{t}$ final states is much higher than that obtained in inclusive *b*-jet measurements at the Tevatron [18] or the LHC [55].

**Table 4:** Purity estimation for *b*-jets in the dilepton channel. The uncertainty on the purity arises from the uncertainties in the signal and background fractions

Process	*α*_*k*_	*p*_*k*_(*b*)
$t\bar{t}$	0.949	0.997
*Z*→*ℓ* ^+^ *ℓ* ^−^	0.006	0.515
Other EW (*W*, diboson)	0.002	0.375
Single top	0.043	0.987
Multi-jet	–	–
**Weighted total**	–	$(99.3^{+0.7}_{-6.5})~\%$

## Jet shapes in the single-lepton channel

For the jet shape calculation, locally calibrated topological clusters are used [54, 57, 58]. In this procedure, effects due to calorimeter response, leakage, and losses in the dead material upstream of the calorimeter are taken into account separately for electromagnetic and hadronic clusters [59].

The differential jet shape *ρ*(*r*) in an annulus of inner radius *r*−*Δr*/2 and outer radius *r*+*Δr*/2 from the axis of a given jet is defined as

2 $$\begin{aligned} \rho(r) = \frac{1}{\varDelta r}\frac{p_{\mathrm{T}}(r-\varDelta r/2,r+\varDelta r/2)}{p_{\mathrm{T}}(0,R)} \end{aligned}$$

Here, *Δr*=0.04 is the width of the annulus; *r*, such that *Δr*/2≤*r*≤*R*−*Δr*/2, is the distance to the jet axis in the *η*-*ϕ* plane, and *p*_T_(*r*_1_,*r*_2_) is the scalar sum of the *p*_T_ of the jet constituents with radii between *r*_1_ and *r*_2_.

**Fig. 4:**
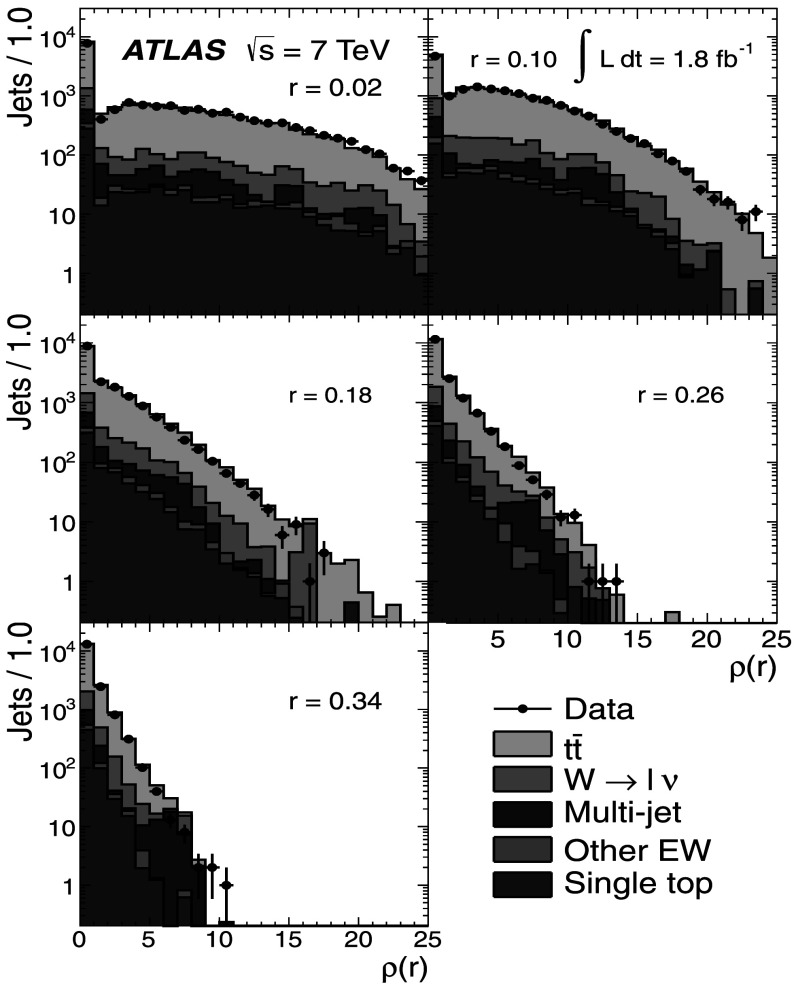
Distribution of *R*=0.4 *b*-jets in the single-lepton channel as a function of the differential jet shapes *ρ*(*r*) for different values of *r*

Some distributions of *ρ*(*r*) are shown in Fig. 4 for the *b*-jet sample selected in the single-lepton channel. There is a marked peak at zero energy deposit, which indicates that energy is concentrated around relatively few particles. As *r* increases, the distributions of *ρ*(*r*) are concentrated at smaller values because of the relatively low energy density at the periphery of the jets. Both effects are well reproduced by the MC generators.

**Fig. 5:**
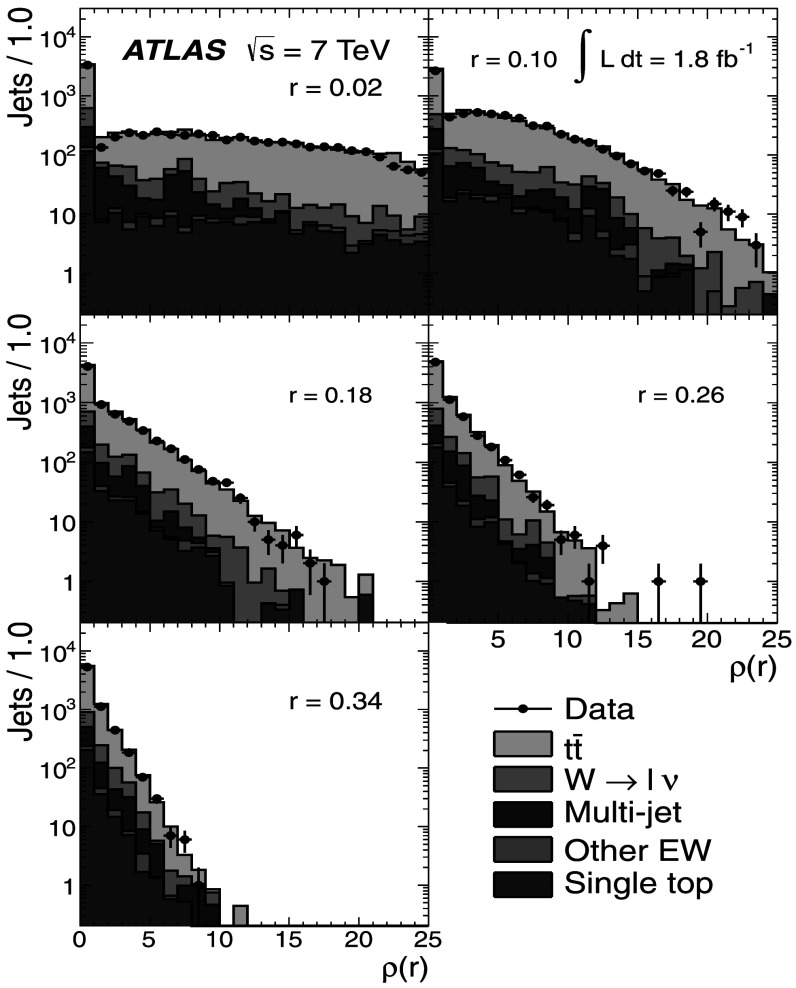
Distribution of *R*=0.4 light jets in the single-lepton channel as a function of the differential jet shapes *ρ*(*r*) for different values of *r*

The analogous *ρ*(*r*) distributions for light jets are shown in Fig. 5. The gross features are similar to those previously discussed for *b*-jets, but for small values of *r*, the *ρ*(*r*) distributions for light jets are somewhat flatter than those for *b*-jets.

**Fig. 6:**
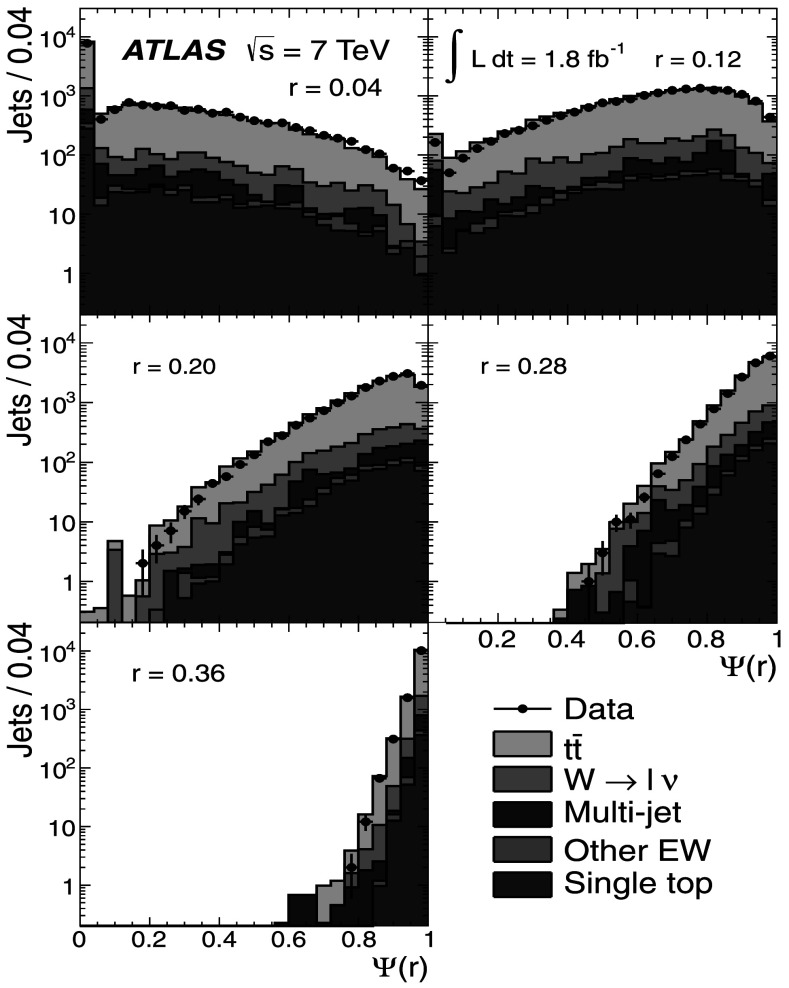
Distribution of *R*=0.4 *b*-jets in the single-lepton channel as a function of the integrated jet shapes *Ψ*(*r*) for different values of *r*

**Fig. 7:**
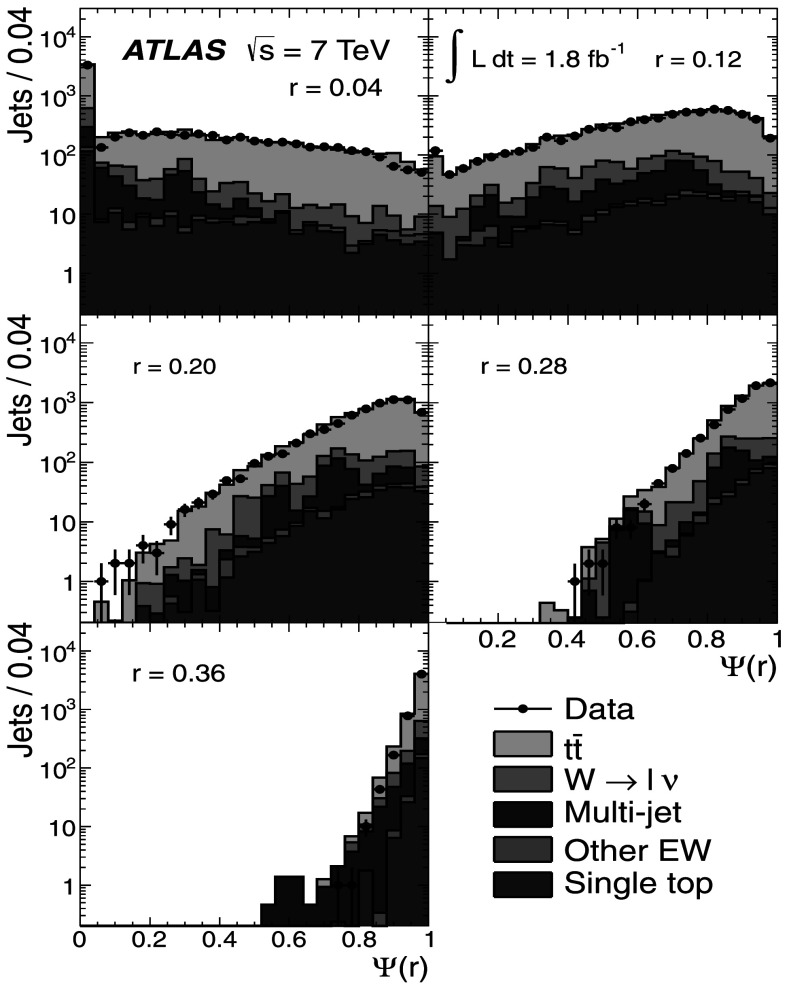
Distribution of *R*=0.4 light jets in the single-lepton channel as a function of the integrated jet shapes *Ψ*(*r*) for different values of *r*

**Fig. 8:**
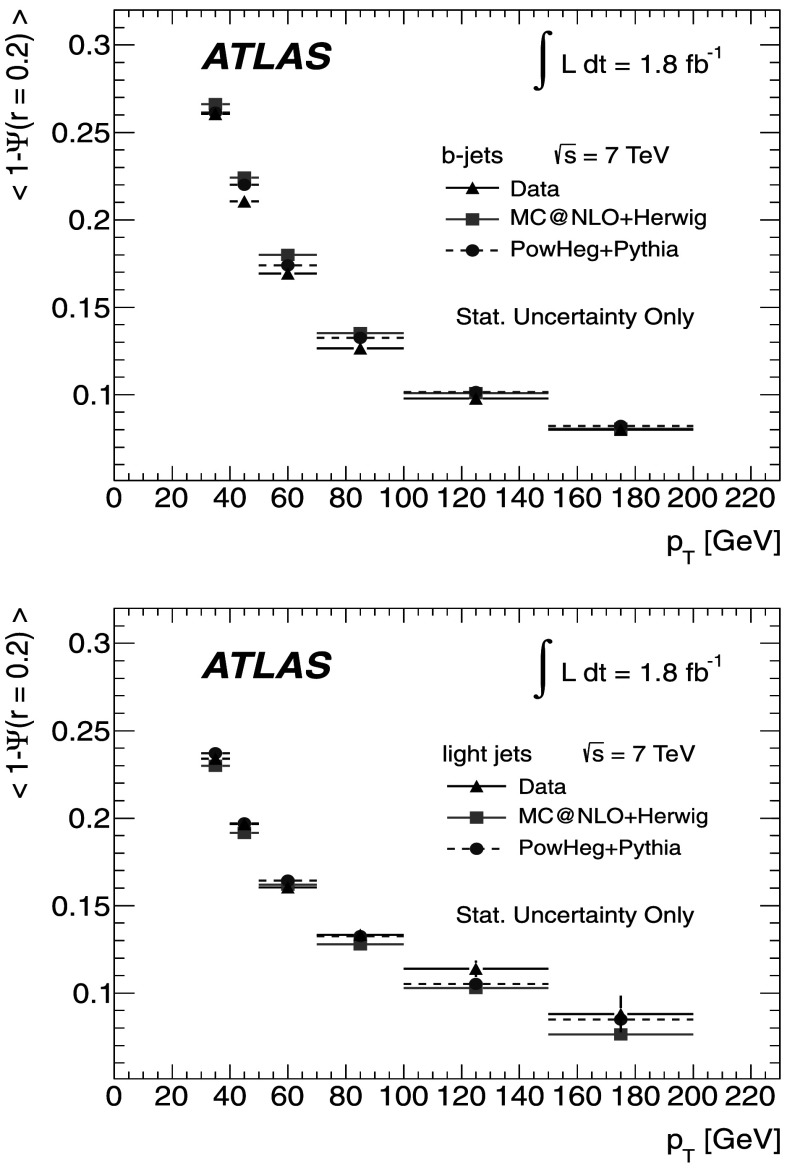
Dependence of the *b*-jet (*top*) and light-jet (*bottom*) shapes on the jet transverse momentum. This dependence is quantified by plotting the mean value 〈1−*Ψ*(*r*=0.2)〉 (the fraction of energy in the outer half of the jet cone) as a function of *p*_T_ for jets in the single-lepton sample

**Fig. 9:**
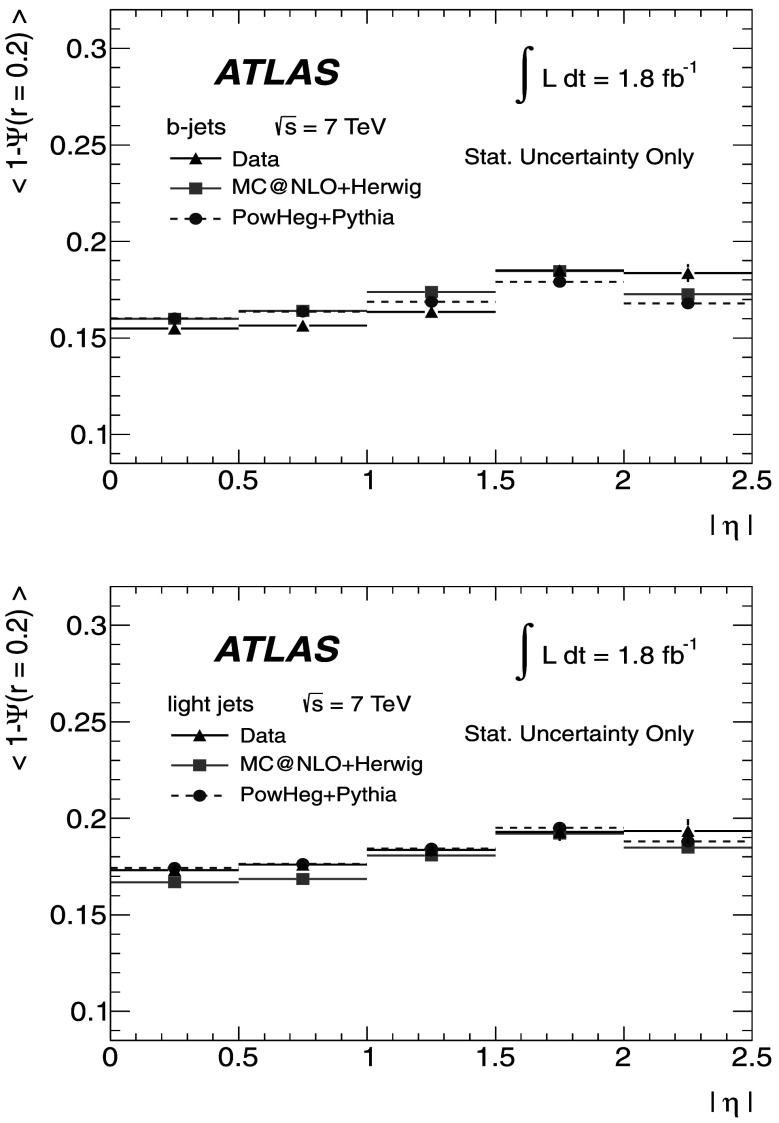
Dependence of the *b*-jet (*top*) and light-jet (*bottom*) shape on the jet pseudorapidity. This dependence is quantified by plotting the mean value 〈1−*Ψ*(*r*=0.2)〉 (the fraction of energy in the outer half of the jet cone) as a function of |*η*| for jets in the single-lepton sample

The integrated jet shape in a cone of radius *r*≤*R* around the jet axis is defined as the cumulative distribution for *ρ*(*r*), i.e.

3 $$\begin{aligned} \varPsi(r) = \frac{p_{\mathrm{T}}(0,r)}{p_{\mathrm{T}}(0,R)}; \quad r \leq R \end{aligned}$$

which satisfies *Ψ*(*r*=*R*)=1. Figure 6 (Fig. 7) shows distributions of the integrated jet shapes for *b*-jets (light jets) in the single-lepton sample. These figures show the inclusive (i.e. not binned in either *η* or *p*_T_) *ρ*(*r*) and *Ψ*(*r*) distributions for fixed values of *r*. Jet shapes are only mildly dependent on pseudorapidity, while they strongly depend on the transverse momentum. This behaviour has been verified in previous analyses [5–11]. This is illustrated in Figs. 8 and 9, which show the energy fraction in the outer half of the cone as a function of *p*_T_ and |*η*|. For this reason, all the data presented in the following are binned in five *p*_T_ regions with *p*_T_<150 GeV, where the statistical uncertainty is small enough. In the following, only the average values of these distributions are presented:

4 $$\begin{aligned} &\bigl\langle \rho(r)\bigr\rangle = \frac{1}{\varDelta r}\frac {1}{N_{\mathrm {jets}}}\sum _{\mathrm{jets}}\frac{p_{\mathrm{T}}(r-\varDelta r/2,r+\varDelta r/2)}{p_{\mathrm{T}}(0,R)} \end{aligned}$$

5 $$\begin{aligned} &\bigl\langle \varPsi(r)\bigr\rangle = \frac{1}{N_{\mathrm {jets}}}\sum _{\mathrm {jets}}\frac{p_{\mathrm{T}}(0,r)}{p_{\mathrm{T}}(0,R)} \end{aligned}$$

where the sum is performed over all jets of a given sample, light jets (*l*) or *b*-jets (*b*) and *N*_jets_ is the number of jets in the sample.

## Results at the detector level

**Fig. 10:**
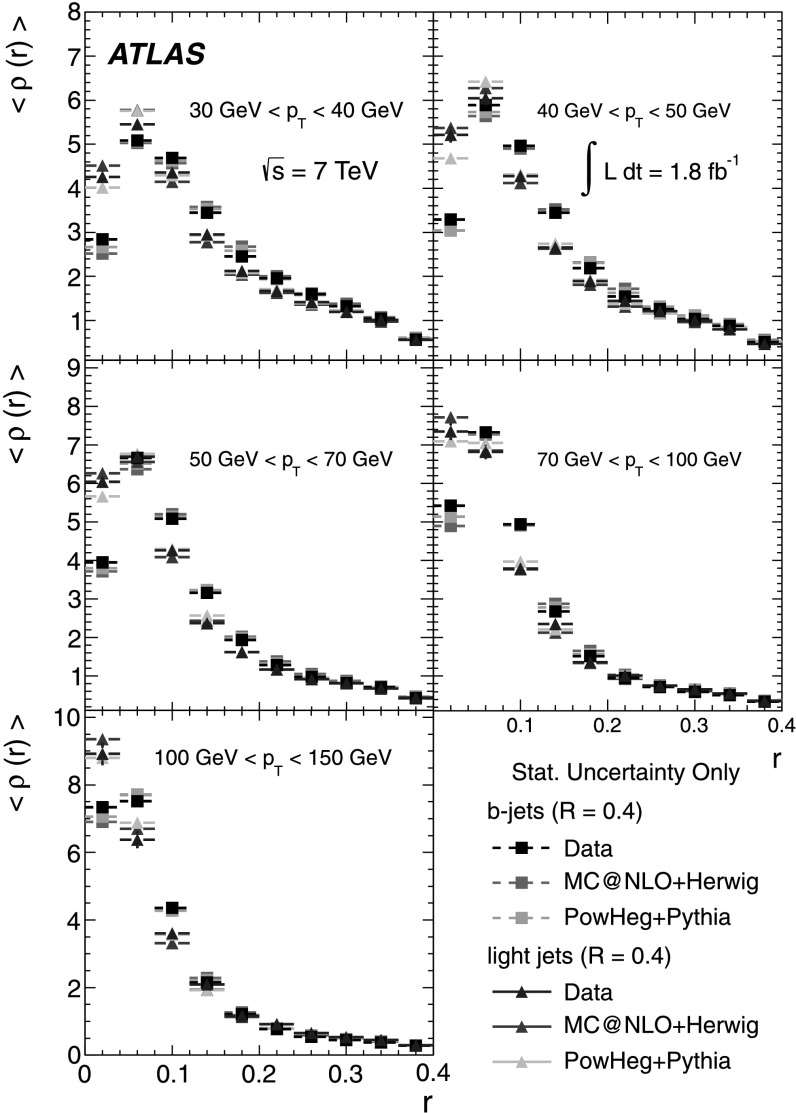
Average values of the differential jet shapes 〈*ρ*(*r*)〉 for light jets (*triangles*) and *b*-jets (*squares*), with *Δr*=0.04, as a function of *r* at the detector level, compared to MC@NLO+Herwig and Powheg+Pythia event generators. The uncertainties shown for data are only statistical

**Fig. 11:**
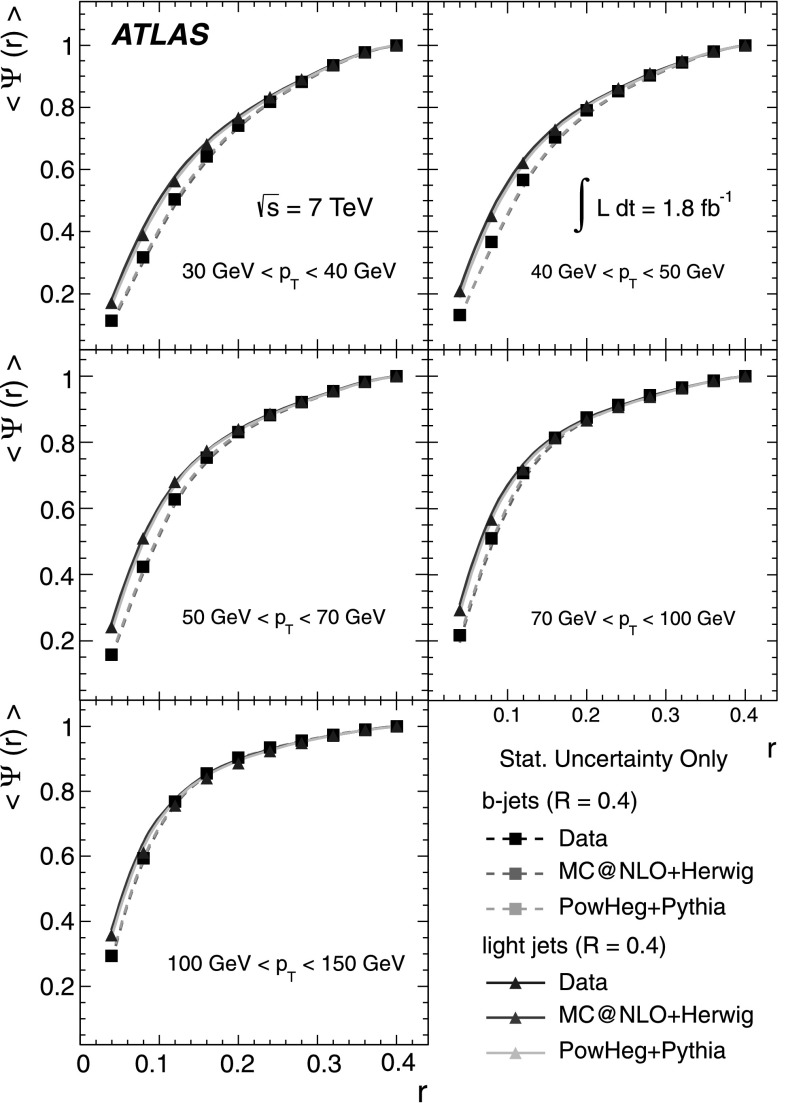
Average values of the integrated jet shapes 〈*Ψ*(*r*)〉 for light jets (*triangles*) and *b*-jets (*squares*), with *Δr*=0.04, as a function of *r* at the detector level, compared to MC@NLO+Herwig and Powheg+Pythia event generators. The uncertainties shown for data are only statistical

In the following, the detector-level results for the average values 〈*ρ*(*r*)〉 and 〈*Ψ*(*r*)〉 as a function of the jet internal radius *r*, are presented. A comparison has been made between *b*-jet shapes obtained in both the dilepton and single-lepton samples, and it is found that they are consistent with each other within the uncertainties. Thus the samples are merged. In Fig. 10, the distributions for the average values of the differential jet shapes are shown for each *p*_T_ bin, along with a comparison with the expectations from the simulated samples described in Sect. 3. There is a small but clear difference between light- and *b*-jet differential shapes, the former lying above (below) the latter for smaller (larger) values of *r*. These differences are more visible at low transverse momentum. In Fig. 11, the average integrated jet shapes 〈*Ψ*(*r*)〉 are shown for both the light jets and *b*-jets, and compared to the MC expectations discussed earlier. Similar comments apply here: The values of 〈*Ψ*(*r*)〉 are consistently larger for light jets than for *b*-jets for small values of *r*, while they tend to merge as *r*→*R* since, by definition, *Ψ*(*R*)=1.

## Unfolding to particle level

In order to correct the data for acceptance and detector effects, thus enabling comparisons with different models and other experiments, an unfolding procedure is followed. The method used to correct the measurements based on topological clusters to the particle level relies on a bin-by-bin correction. Correction factors *F*(*r*) are calculated separately for differential, 〈*ρ*(*r*)〉, and integrated, 〈*Ψ*(*r*)〉, jet shapes in both the light- and *b*-jet samples. For differential (*ρ*) and integrated jet shapes (*Ψ*), they are defined as the ratio of the particle-level quantity to the detector-level quantity as described by the MC simulations discussed in Sect. 3, i.e.

6 $$\begin{aligned} &F^{\rho}_{\mathrm{l},b}(r) = \frac{\langle\rho(r)_{\mathrm {l},b}\rangle _{\mathrm{MC,part}}}{\langle\rho(r)_{\mathrm{l},b}\rangle _{\mathrm {MC,det}}} \end{aligned}$$

7 $$\begin{aligned} &F^{\varPsi}_{\mathrm{l},b}(r) = \frac{\langle\varPsi(r)_{\mathrm {l},b}\rangle _{\mathrm{MC,part}}}{\langle\varPsi(r)_{\mathrm{l},b}\rangle _{\mathrm{MC,det}}} \end{aligned}$$

While the detector-level MC includes the background sources described before, the particle-level jets are built using all particles in the signal sample with an average lifetime above 10^−11^ s, excluding muons and neutrinos. The results have only a small sensitivity to the inclusion or not of muons and neutrinos, as well as to the background estimation. For particle-level *b*-jets, a *b*-hadron with *p*_T_>5 GeV is required to be closer than *ΔR*=0.3 from the jet axis, while for light jets, a selection equivalent to that for the detector-level jets is applied, selecting the non-*b*-jet pair with invariant mass closest to *m*_*W*_. The same kinematic selection criteria are applied to these particle-level jets as for the reconstructed jets, namely *p*_T_>25 GeV, |*η*|<2.5 and *ΔR*>0.8 to avoid jet–jet overlaps.

A Bayesian iterative unfolding approach [64] is used as a cross-check. The RooUnfold software [65] is used by providing the jet-by-jet information on the jet shapes, in the *p*_T_ intervals defined above. This method takes into account bin-by-bin migrations in the *ρ*(*r*) and *Ψ*(*r*) distributions for fixed values of *r*. The results of the bin-by-bin and the Bayesian unfolding procedures agree at the 2 % level.

As an additional check of the stability of the unfolding procedure, the directly unfolded integrated jet shapes are compared with those obtained from integrating the unfolded differential distributions. The results agree to better than 1 %. These results are reassuring since the differential and integrated jet shapes are subject to migration and resolution effects in different ways. Both quantities are also subject to bin-to-bin correlations. For the differential measurement, the correlations arise from the common normalisation. They increase with the jet transverse momentum, varying from 25 % to 50 % at their maximum, which is reached for neighbouring bins at low *r*. The correlations for the integrated measurement are greater and their maximum varies from 60 % to 75 % as the jet *p*_T_ increases.

## Systematic uncertainties

The main sources of systematic uncertainty are described below.

- The energy of individual clusters inside the jet is varied according to studies using isolated tracks [67], parameterising the uncertainty on the calorimeter energy measurements as a function of the cluster *p*_T_. The impact on the differential jet shape increases from 2 % to 10 % as the edge of the jet cone is approached.
- The coordinates *η*, *ϕ* of the clusters are smeared using a Gaussian distribution with an RMS width of 5 mrad accounting for small differences in the cluster position between data and Monte Carlo [66]. This smearing has an effect on the jet shape which is smaller than 2 %.
- An uncertainty arising from the amount of passive material in the detector is derived using the algorithm described in Ref. [66] as a result of the studies carried out in Ref. [67]. Low-energy clusters (*E*<2.5 GeV) are removed from the reconstruction according to a probability function given by $\mathcal{P}(E=0)\times\mathrm{e}^{-2E}$, where $\mathcal{P}(E = 0)$ is the measured probability (28 %) of a charged particle track to be associated with a zero energy deposit in the calorimeter and *E* is the cluster energy in GeV. As a result, approximately 6 % of the total number of clusters are discarded. The impact of this cluster-removing algorithm on the measured jet shapes is smaller than 2 %.
- As a further cross-check an unfolding of the track-based jet shapes to the particle level has also been performed. The differences from those obtained using calorimetric measurements are of a similar scale to the ones discussed for the cluster energy, angular smearing and dead material.
- An uncertainty arising from the jet energy calibration (JES) is taken into account by varying the jet energy scale in the range 2 % to 8 % of the measured value, depending on the jet *p*_T_ and *η*. This variation is different for light jets and *b*-jets since they have a different particle composition.
- The jet energy resolution is also taken into account by smearing the jet *p*_T_ using a Gaussian centred at unity and with standard deviation *σ*_r_ [68]. The impact on the measured jet shapes is about 5 %.
- The uncertainty due to the JVF requirement is estimated by comparing the jet shapes with and without this requirement. The uncertainty is smaller than 1 %.
- An uncertainty is also assigned to take pile-up effects into account. This is done by calculating the differences between samples where the number of *pp* interaction vertices is smaller (larger) than five and the total sample. The impact on the differential jet shapes varies from 2 % to 10 % as *r* increases.
- An additional uncertainty due to the unfolding method is determined by comparing the correction factors obtained with three different MC samples, Powheg+Pythia, Powheg+Jimmy and AcerMC [35] with the Perugia 2010 tune [36], to the nominal correction factors from the MC@NLO sample. The uncertainty is defined as the maximum deviation of these three unfolding results, and it varies from 1 % to 8 %.

Additional systematic uncertainties associated with details of the analysis such as the working point of the *b*-tagging algorithm and the *ΔR*>0.8 cut between jets, as well as those related to physics object reconstruction efficiencies and variations in the background normalisation are found to be negligible. All sources of systematic uncertainty are propagated through the unfolding procedure. The resulting systematic uncertainties on each differential or integrated shape are added in quadrature. In the case of differential jet shapes, the uncertainty varies from 1 % to 20 % in each *p*_T_ bin as *r* increases, while the uncertainty for the integrated shapes decreases from 10 % to 0 % as one approaches the edge of the jet cone, where *r*=*R*.

## Discussion of the results

**Fig. 12:**
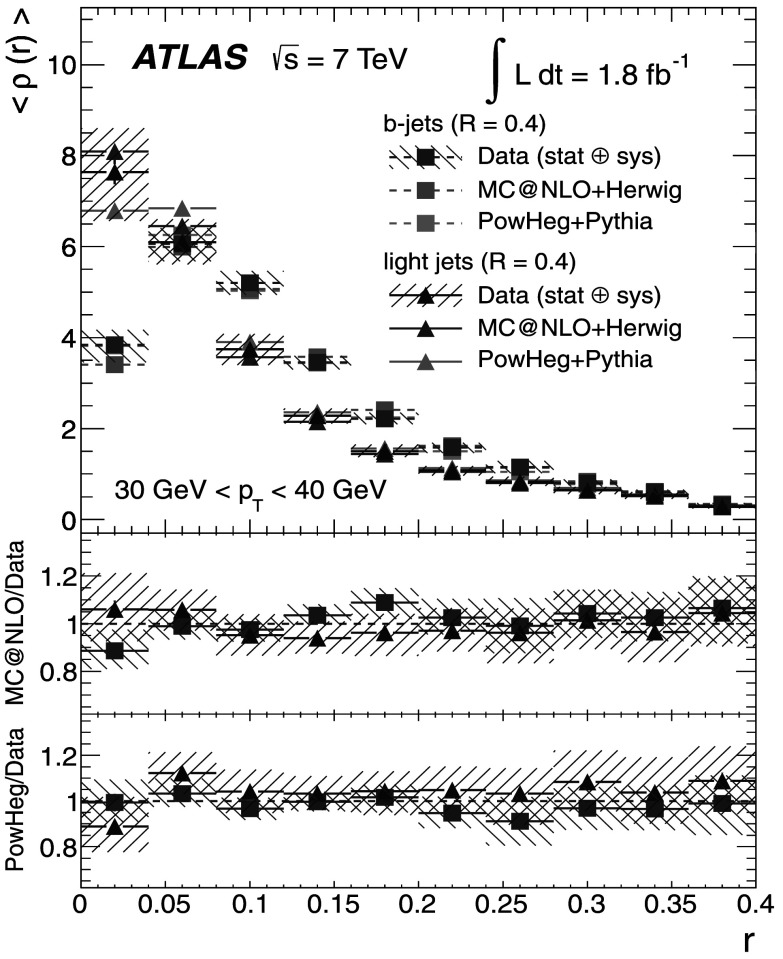
Differential jet shapes 〈*ρ*(*r*)〉 as a function of the radius *r* for light jets (*triangles*) and *b*-jets (*squares*). The data are compared to MC@NLO+Herwig and Powheg+Pythia event generators for 30 GeV<*p*_T_<40 GeV. The uncertainties shown include statistical and systematic sources, added in quadrature

**Fig. 13:**
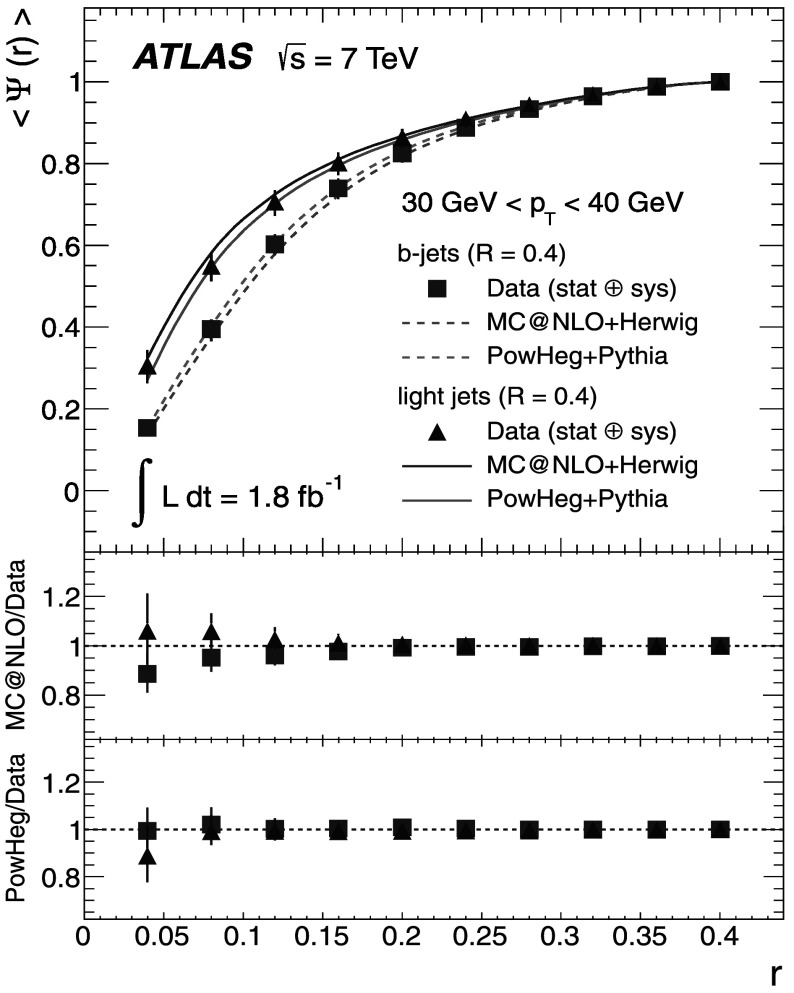
Integrated jet shapes 〈*Ψ*(*r*)〉 as a function of the radius *r* for light jets (*triangles*) and *b*-jets (*squares*). The data are compared to MC@NLO+Herwig and Powheg+Pythia event generators for 30 GeV<*p*_T_<40 GeV. The uncertainties shown include statistical and systematic sources, added in quadrature

**Fig. 14:**
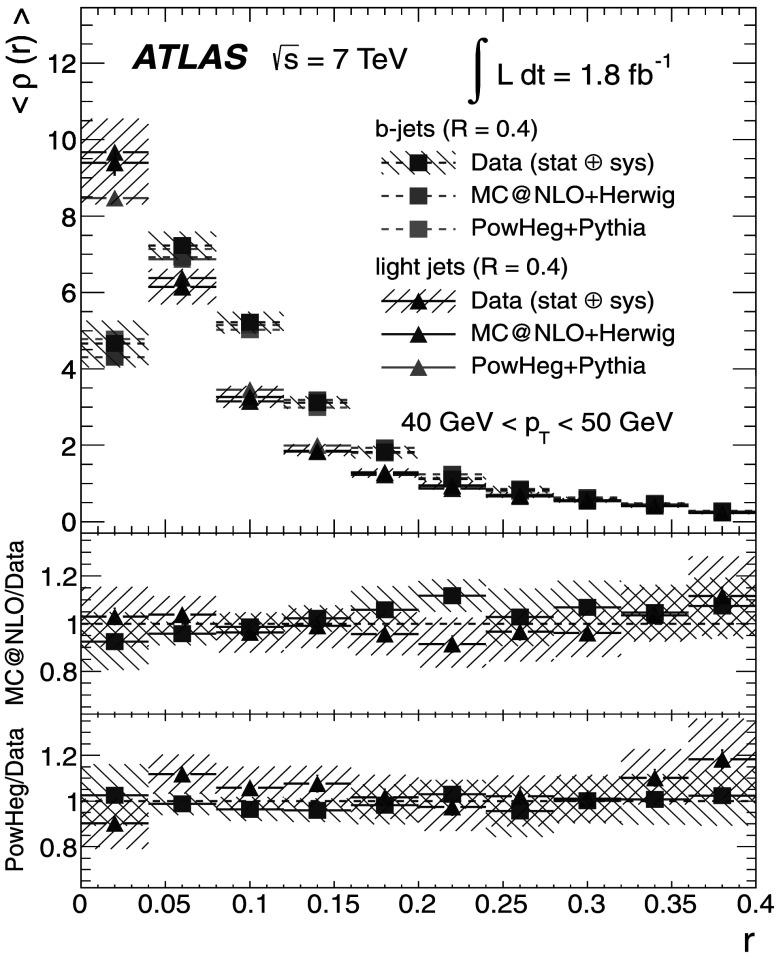
Differential jet shapes 〈*ρ*(*r*)〉 as a function of the radius *r* for light jets (*triangles*) and *b*-jets (*squares*). The data are compared to MC@NLO+Herwig and Powheg+Pythia event generators for 40 GeV<*p*_T_<50 GeV. The uncertainties shown include statistical and systematic sources, added in quadrature

**Fig. 15:**
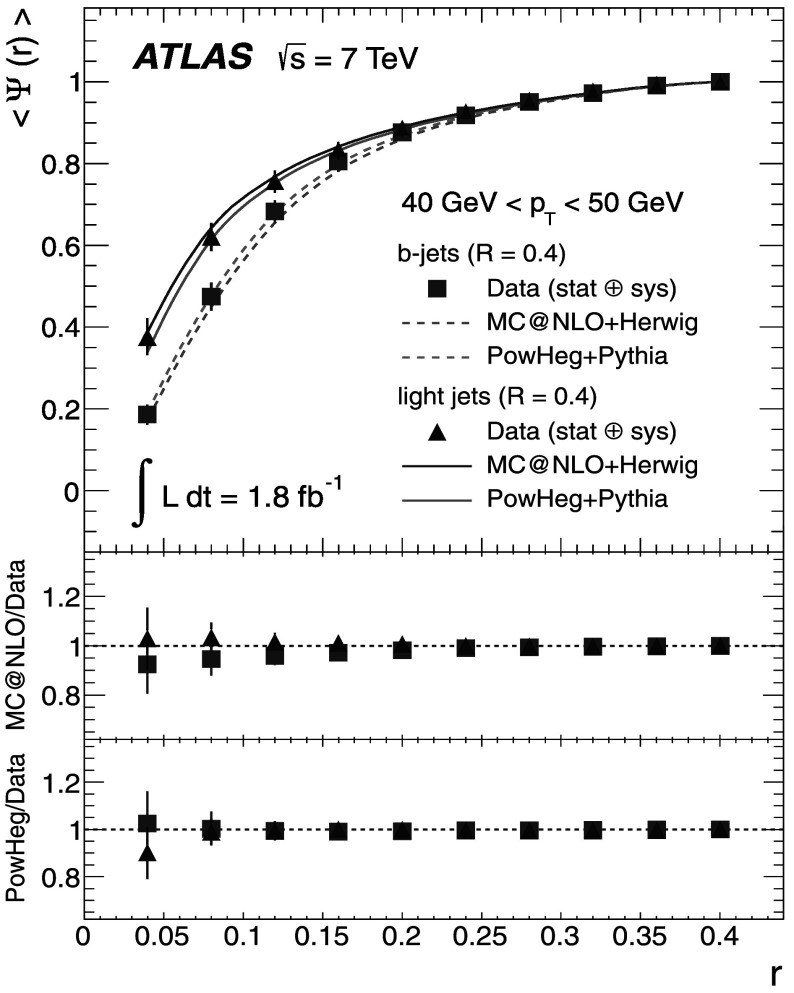
Integrated jet shapes 〈*Ψ*(*r*)〉 as a function of the radius *r* for light jets (*triangles*) and *b*-jets (*squares*). The data are compared to MC@NLO+Herwig and Powheg+Pythia event generators for 40 GeV<*p*_T_<50 GeV. The uncertainties shown include statistical and systematic sources, added in quadrature

**Fig. 16:**
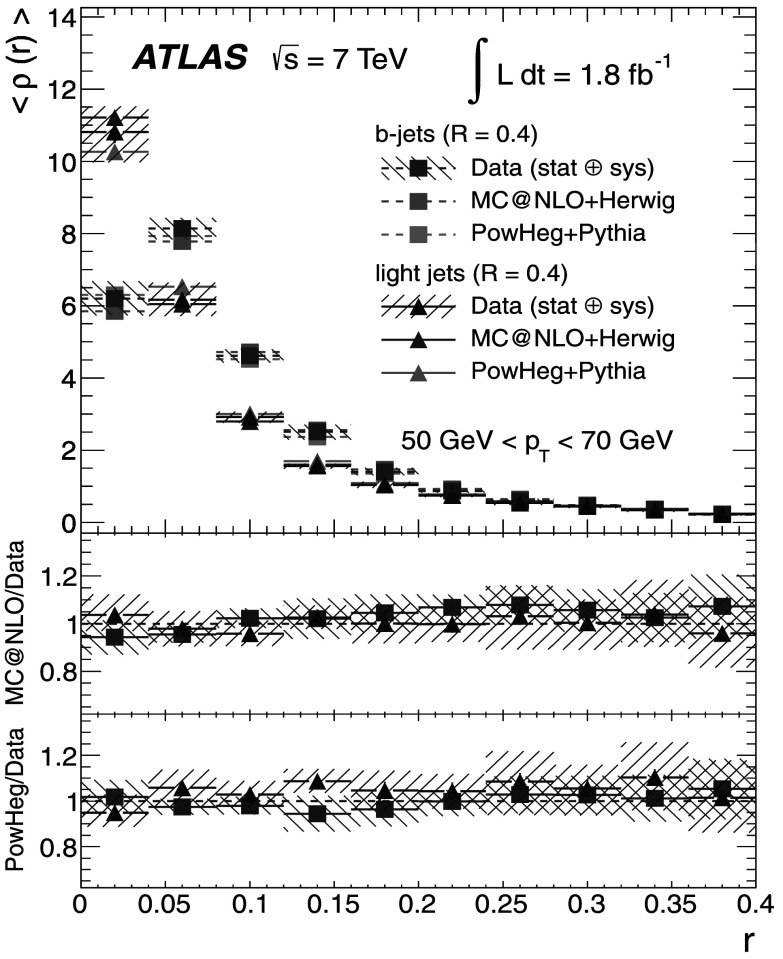
Differential jet shapes 〈*ρ*(*r*)〉 as a function of the radius *r* for light jets (*triangles*) and *b*-jets (*squares*). The data are compared to MC@NLO+Herwig and Powheg+Pythia event generators for 50 GeV<*p*_T_<70 GeV. The uncertainties shown include statistical and systematic sources, added in quadrature

**Fig. 17:**
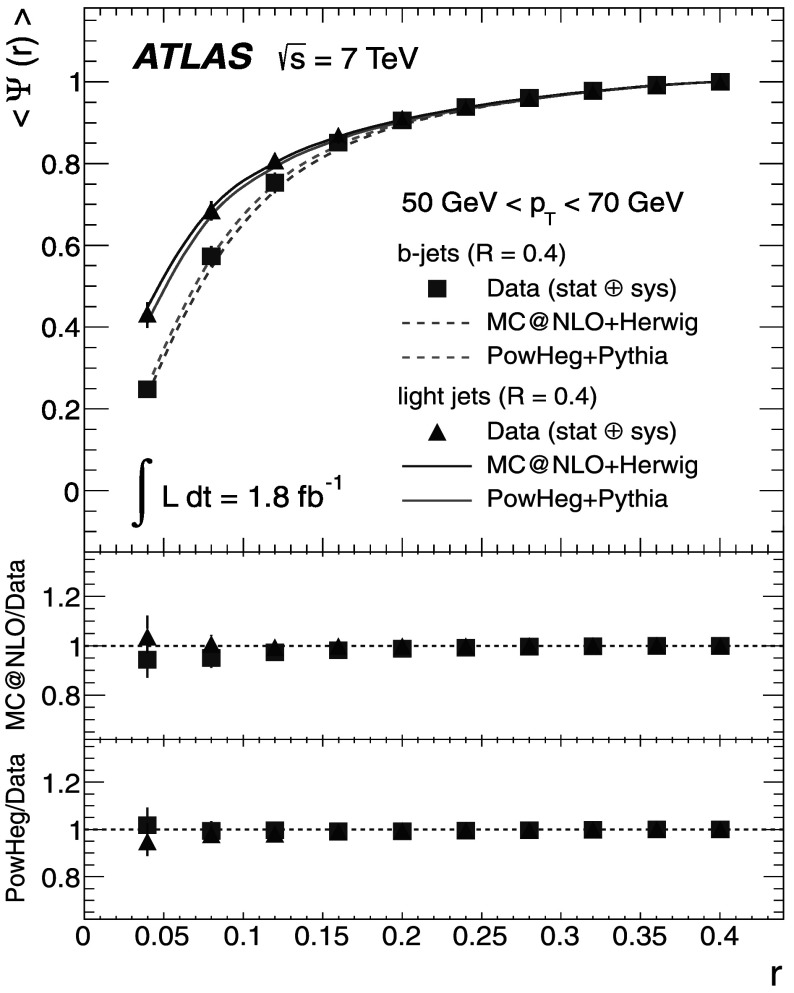
Integrated jet shapes 〈*Ψ*(*r*)〉 as a function of the radius *r* for light jets (*triangles*) and *b*-jets (*squares*). The data are compared to MC@NLO+Herwig and Powheg+Pythia event generators for 50 GeV<*p*_T_<70 GeV. The uncertainties shown include statistical and systematic sources, added in quadrature

**Fig. 18:**
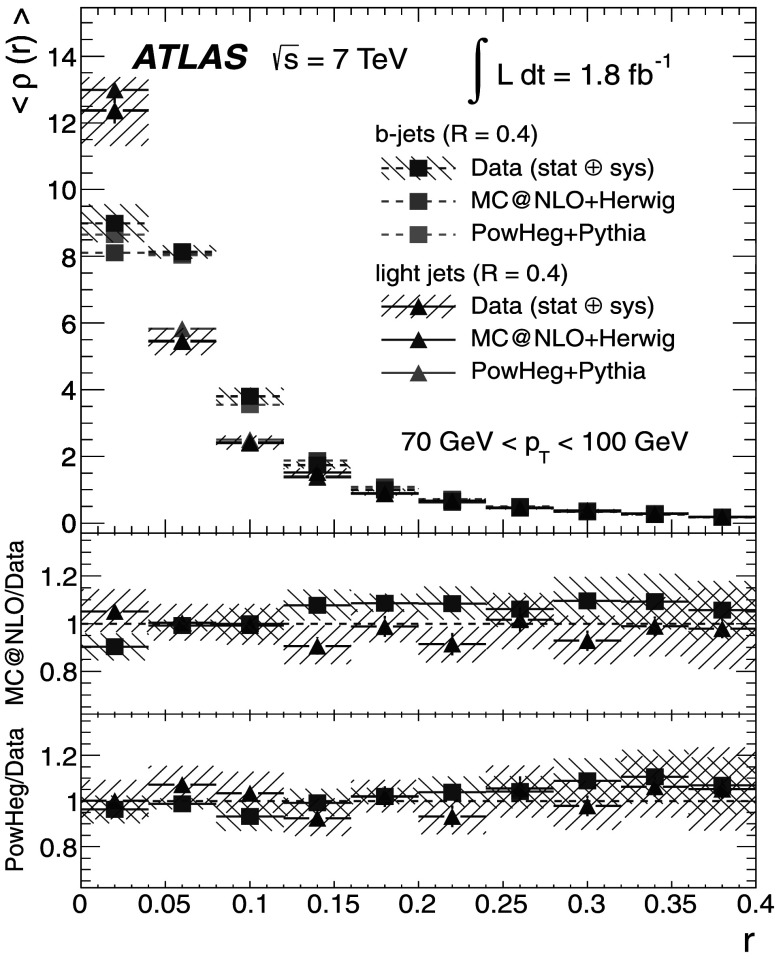
Differential jet shapes 〈*ρ*(*r*)〉 as a function of the radius *r* for light jets (*triangles*) and *b*-jets (*squares*). The data are compared to MC@NLO+Herwig and Powheg+Pythia event generators for 70 GeV<*p*_T_<100 GeV. The uncertainties shown include statistical and systematic sources, added in quadrature

**Fig. 19:**
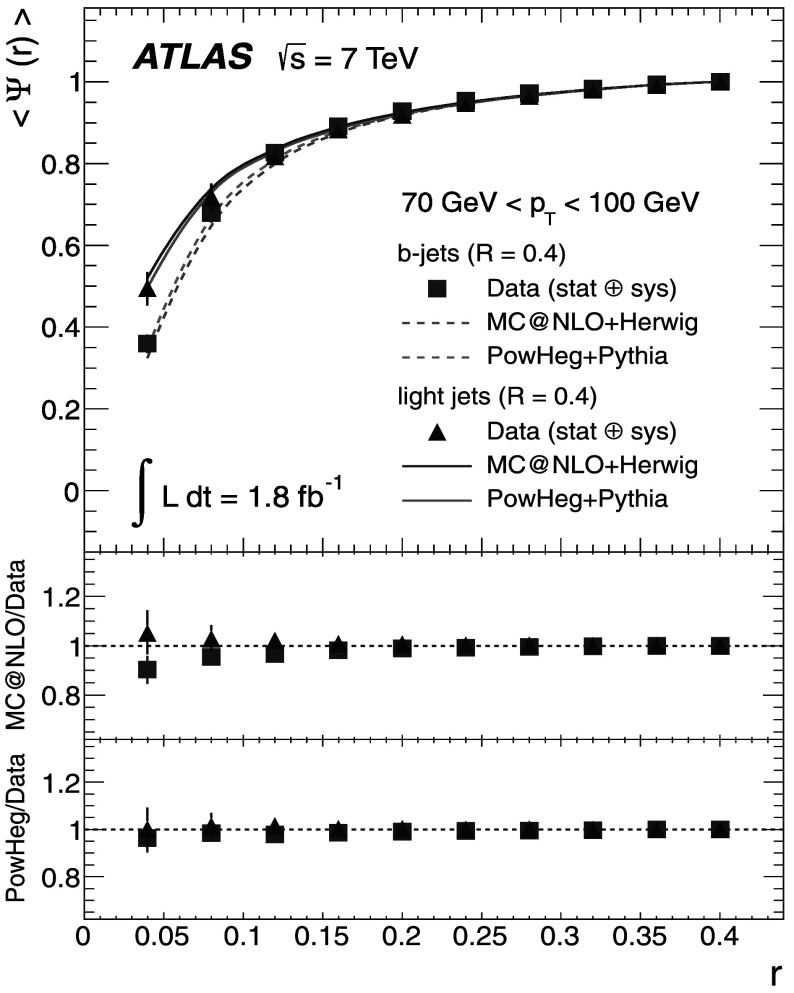
Integrated jet shapes 〈*Ψ*(*r*)〉 as a function of the radius *r* for light jets (*triangles*) and *b*-jets (*squares*). The data are compared to MC@NLO+Herwig and Powheg+Pythia event generators for 70 GeV<*p*_T_<100 GeV. The uncertainties shown include statistical and systematic sources, added in quadrature

**Fig. 20:**
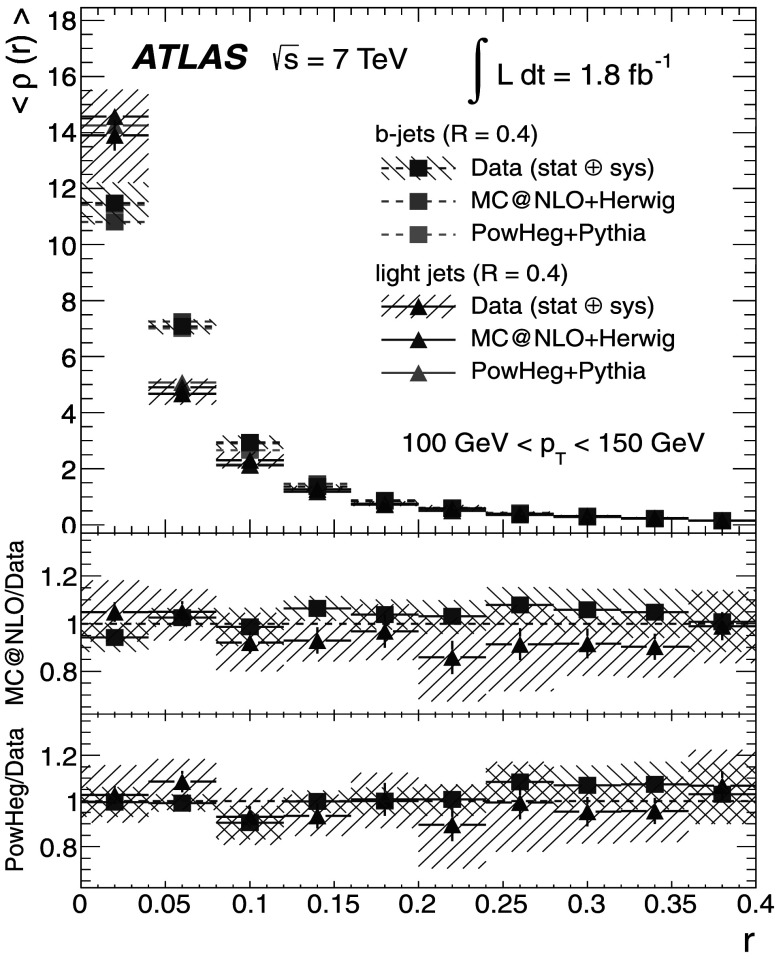
Differential jet shapes 〈*ρ*(*r*)〉 as a function of the radius *r* for light jets (*triangles*) and *b*-jets (*squares*). The data are compared to MC@NLO+Herwig and Powheg+Pythia event generators for 100 GeV<*p*_T_<150 GeV. The uncertainties shown include statistical and systematic sources, added in quadrature

**Fig. 21:**
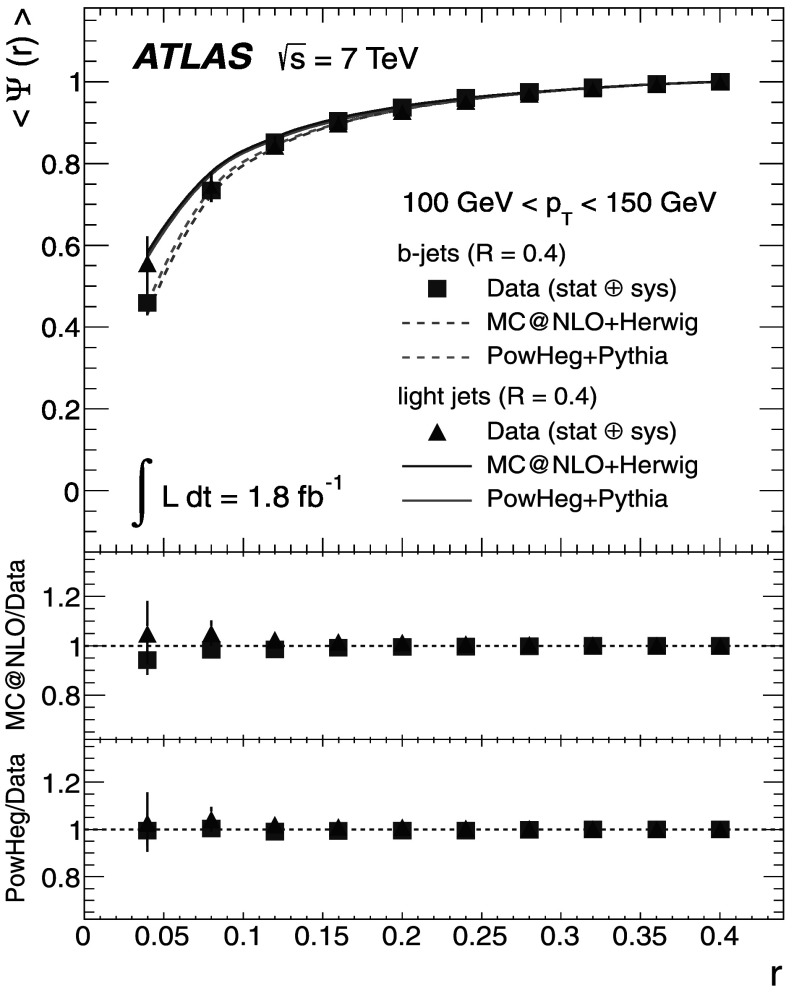
Integrated jet shapes 〈*Ψ*(*r*)〉 as a function of the radius *r* for light jets (*triangles*) and *b*-jets (*squares*). The data are compared to MC@NLO+Herwig and Powheg+Pythia event generators for 100 GeV<*p*_T_<150 GeV. The uncertainties shown include statistical and systematic sources, added in quadrature

The results at the particle level are presented, together with the total uncertainties arising from statistical and systematic effects. The averaged differential jet shapes 〈*ρ*(*r*)〉 are shown in the even-numbered Figs. 12–20 as a function of *r* and in bins of *p*_T_, while numerical results are presented in the odd-numbered Tables 5–13. The observation made at the detector level in Sect. 8 that *b*-jets are broader than light jets is strengthened after unfolding because it also corrects the light-jet sample for purity effects. Similarly, the odd-numbered Figs. 13–21 show the integrated shapes 〈*Ψ*(*r*)〉 as a function of *r* and in bins of *p*_T_ for light jets and *b*-jets. Numerical results are presented in the even-numbered Tables 6–14. As before, the observation is made that *b*-jets have a wider energy distribution inside the jet cone than light jets, as it can be seen that 〈*Ψ*_*b*_〉<〈*Ψ*_l_〉 for low *p*_T_ and small *r*.

**Table 5:** Unfolded values for 〈*ρ*(*r*)〉, together with statistical and systematic uncertainties for 30 GeV<*p*_T_<40 GeV

*r*	〈*ρ*_*b*_(*r*)〉 [*b*-jets]	〈*ρ*_l_(*r*)〉 [light jets]
0.02	$3.84 \pm0.15 ^{+ 0.29 }_{- 0.36 }$	$7.64 \pm0.27 ^{+ 0.93 }_{- 1.10 }$
0.06	$6.06 \pm0.14 ^{+ 0.31 }_{- 0.36 }$	$6.10 \pm0.16 ^{+ 0.48 }_{- 0.47 }$
0.10	$5.20 \pm0.11 ^{+ 0.24 }_{- 0.23 }$	$3.75 \pm0.10 ^{+ 0.32 }_{- 0.33 }$
0.14	$3.45 \pm0.09 ^{+ 0.12 }_{- 0.13 }$	$2.28 \pm0.07 ^{+ 0.14 }_{- 0.16 }$
0.18	$2.21 \pm0.06 ^{+ 0.13 }_{- 0.11 }$	$1.50 \pm0.05 ^{+ 0.14 }_{- 0.12 }$
0.22	$1.58 \pm0.04 ^{+ 0.10 }_{- 0.11 }$	$1.08 \pm0.03 ^{+ 0.09 }_{- 0.10 }$
0.26	$1.15 \pm0.03 ^{+ 0.13 }_{- 0.13 }$	$0.83 \pm0.03 ^{+ 0.11 }_{- 0.09 }$
0.30	$0.80 \pm0.02 ^{+ 0.08 }_{- 0.07 }$	$0.64 \pm0.02 ^{+ 0.07 }_{- 0.08 }$
0.34	$0.60 \pm0.01 ^{+ 0.06 }_{- 0.06 }$	$0.53 \pm0.01 ^{+ 0.07 }_{- 0.08 }$
0.38	$0.32 \pm0.01 ^{+ 0.04 }_{- 0.04 }$	$0.28 \pm0.01 ^{+ 0.04 }_{- 0.04 }$

**Table 6:** Unfolded values for 〈*Ψ*(*r*)〉, together with statistical and systematic uncertainties for 30 GeV<*p*_T_<40 GeV

*r*	〈*Ψ*_*b*_(*r*)〉 [*b*-jets]	〈*Ψ*_l_(*r*)〉 [light jets]
0.04	$0.154 \pm0.006 ^{+ 0.012 }_{- 0.014 }$	$0.306 \pm0.011 ^{+ 0.037 }_{- 0.043 }$
0.08	$0.395 \pm0.007 ^{+ 0.023 }_{- 0.028 }$	$0.550 \pm0.009 ^{+ 0.031 }_{- 0.037 }$
0.12	$0.602 \pm0.006 ^{+ 0.025 }_{- 0.026 }$	$0.706 \pm0.007 ^{+ 0.028 }_{- 0.034 }$
0.16	$0.739 \pm0.004 ^{+ 0.025 }_{- 0.025 }$	$0.802 \pm0.005 ^{+ 0.025 }_{- 0.030 }$
0.20	$0.825 \pm0.003 ^{+ 0.020 }_{- 0.023 }$	$0.863 \pm0.004 ^{+ 0.020 }_{- 0.025 }$
0.24	$0.887 \pm0.003 ^{+ 0.016 }_{- 0.017 }$	$0.907 \pm0.003 ^{+ 0.016 }_{- 0.019 }$
0.28	$0.934 \pm0.002 ^{+ 0.012 }_{- 0.012 }$	$0.942 \pm0.002 ^{+ 0.011 }_{- 0.014 }$
0.32	$0.964 \pm0.001 ^{+ 0.007 }_{- 0.007 }$	$0.967 \pm0.001 ^{+ 0.007 }_{- 0.008 }$
0.36	$0.988 \pm0.001 ^{+ 0.004 }_{- 0.002 }$	$0.989 \pm0.001 ^{+ 0.003 }_{- 0.003 }$
0.40	1.000	1.000

**Table 7:** Unfolded values for 〈*ρ*(*r*)〉, together with statistical and systematic uncertainties for 40 GeV<*p*_T_<50 GeV

*r*	〈*ρ*_*b*_(*r*)〉 [*b*-jets]	〈*ρ*_l_(*r*)〉 [light jets]
0.02	$4.66 \pm0.15 ^{+ 0.58 }_{- 0.61 }$	$9.39 \pm0.34 ^{+ 1.10 }_{- 1.10 }$
0.06	$7.23 \pm0.14 ^{+ 0.33 }_{- 0.35 }$	$6.14 \pm0.17 ^{+ 0.44 }_{- 0.43 }$
0.10	$5.22 \pm0.11 ^{+ 0.25 }_{- 0.28 }$	$3.27 \pm0.10 ^{+ 0.27 }_{- 0.27 }$
0.14	$3.12 \pm0.07 ^{+ 0.15 }_{- 0.15 }$	$1.85 \pm0.07 ^{+ 0.16 }_{- 0.12 }$
0.18	$1.83 \pm0.05 ^{+ 0.15 }_{- 0.17 }$	$1.28 \pm0.05 ^{+ 0.11 }_{- 0.11 }$
0.22	$1.12 \pm0.03 ^{+ 0.06 }_{- 0.06 }$	$0.95 \pm0.04 ^{+ 0.10 }_{- 0.11 }$
0.26	$0.83 \pm0.02 ^{+ 0.10 }_{- 0.09 }$	$0.69 \pm0.03 ^{+ 0.08 }_{- 0.05 }$
0.30	$0.59 \pm0.02 ^{+ 0.06 }_{- 0.06 }$	$0.56 \pm0.02 ^{+ 0.05 }_{- 0.05 }$
0.34	$0.46 \pm0.01 ^{+ 0.05 }_{- 0.05 }$	$0.41 \pm0.01 ^{+ 0.04 }_{- 0.04 }$
0.38	$0.26 \pm0.01 ^{+ 0.03 }_{- 0.03 }$	$0.23 \pm0.01 ^{+ 0.03 }_{- 0.03 }$

**Table 8:** Unfolded values for 〈*Ψ*(*r*)〉, together with statistical and systematic uncertainties for 40 GeV<*p*_T_<50 GeV

*r*	〈*Ψ*_*b*_(*r*)〉 [*b*-jets]	〈*Ψ*_l_(*r*)〉 [light jets]
0.04	$0.187 \pm0.006 ^{+ 0.023 }_{- 0.024 }$	$0.376 \pm0.013 ^{+ 0.044 }_{- 0.043 }$
0.08	$0.475 \pm0.007 ^{+ 0.033 }_{- 0.034 }$	$0.621 \pm0.011 ^{+ 0.032 }_{- 0.034 }$
0.12	$0.683 \pm0.005 ^{+ 0.027 }_{- 0.029 }$	$0.757 \pm0.008 ^{+ 0.025 }_{- 0.027 }$
0.16	$0.805 \pm0.004 ^{+ 0.023 }_{- 0.025 }$	$0.832 \pm0.006 ^{+ 0.021 }_{- 0.022 }$
0.20	$0.876 \pm0.003 ^{+ 0.017 }_{- 0.018 }$	$0.885 \pm0.004 ^{+ 0.017 }_{- 0.018 }$
0.24	$0.918 \pm0.002 ^{+ 0.015 }_{- 0.016 }$	$0.925 \pm0.003 ^{+ 0.012 }_{- 0.014 }$
0.28	$0.950 \pm0.002 ^{+ 0.010 }_{- 0.011 }$	$0.953 \pm0.002 ^{+ 0.010 }_{- 0.011 }$
0.32	$0.973 \pm0.001 ^{+ 0.007 }_{- 0.006 }$	$0.976 \pm0.001 ^{+ 0.006 }_{- 0.006 }$
0.36	$0.990 \pm0.001 ^{+ 0.003 }_{- 0.002 }$	$0.992 \pm0.001 ^{+ 0.003 }_{- 0.003 }$
0.40	1.000	1.000

**Table 9:** Unfolded values for 〈*ρ*(*r*)〉, together with statistical and systematic uncertainties for 50 GeV<*p*_T_<70 GeV

*r*	〈*ρ*_*b*_(*r*)〉 [*b*-jets]	〈*ρ*_l_(*r*)〉 [light jets]
0.02	$6.19 \pm0.13 ^{+ 0.46 }_{- 0.44 }$	$10.82\pm0.31 ^{+0.64 }_{- 0.84 }$
0.06	$8.14 \pm0.11 ^{+ 0.27 }_{- 0.29 }$	$6.17 \pm0.14 ^{+ 0.45}_{- 0.44 }$
0.10	$4.62 \pm0.06 ^{+ 0.17 }_{- 0.18 }$	$2.92 \pm0.08 ^{+ 0.14}_{- 0.15 }$
0.14	$2.50 \pm0.04 ^{+ 0.20 }_{- 0.21 }$	$1.56 \pm0.05 ^{+ 0.05}_{- 0.06 }$
0.18	$1.40 \pm0.03 ^{+ 0.11 }_{- 0.10 }$	$1.04 \pm0.04 ^{+ 0.08}_{- 0.08 }$
0.22	$0.87 \pm0.02 ^{+ 0.05 }_{- 0.04 }$	$0.75 \pm0.03 ^{+ 0.05}_{- 0.05 }$
0.26	$0.60 \pm0.01 ^{+ 0.05 }_{- 0.04 }$	$0.54 \pm0.02 ^{+ 0.07}_{- 0.06 }$
0.30	$0.45 \pm0.01 ^{+ 0.04 }_{- 0.04 }$	$0.44 \pm0.01 ^{+ 0.05}_{- 0.04 }$
0.34	$0.36 \pm0.01 ^{+ 0.04 }_{- 0.04 }$	$0.34 \pm0.01 ^{+ 0.04}_{- 0.05 }$
0.38	$0.21 \pm0.00 ^{+ 0.03 }_{- 0.03 }$	$0.23 \pm0.01 ^{+ 0.03}_{- 0.04 }$

**Table 10:** Unfolded values for 〈*Ψ*(*r*)〉, together with statistical and systematic uncertainties for 50 GeV<*p*_T_<70 GeV

*r*	〈*Ψ*_*b*_(*r*)〉 [*b*-jets]	〈*Ψ*_l_(*r*)〉 [light jets]
0.04	$0.248 \pm0.005 ^{+ 0.019 }_{- 0.018 }$	$0.433 \pm0.012 ^{+ 0.026 }_{- 0.034 }$
0.08	$0.573 \pm0.005 ^{+ 0.024 }_{- 0.023 }$	$0.686 \pm0.009 ^{+ 0.020 }_{- 0.024 }$
0.12	$0.753 \pm0.004 ^{+ 0.025 }_{- 0.025 }$	$0.807 \pm0.006 ^{+ 0.017 }_{- 0.019 }$
0.16	$0.851 \pm0.003 ^{+ 0.019 }_{- 0.018 }$	$0.868 \pm0.004 ^{+ 0.017 }_{- 0.019 }$
0.20	$0.905 \pm0.002 ^{+ 0.015 }_{- 0.015 }$	$0.909 \pm0.003 ^{+ 0.014 }_{- 0.016 }$
0.24	$0.938 \pm0.001 ^{+ 0.012 }_{- 0.013 }$	$0.939 \pm0.002 ^{+ 0.012 }_{- 0.014 }$
0.28	$0.961 \pm0.001 ^{+ 0.008 }_{- 0.009 }$	$0.960 \pm0.002 ^{+ 0.008 }_{- 0.009 }$
0.32	$0.978 \pm0.001 ^{+ 0.005 }_{- 0.005 }$	$0.977 \pm0.001 ^{+ 0.006 }_{- 0.006 }$
0.36	$0.992 \pm0.000 ^{+ 0.003 }_{- 0.002 }$	$0.990 \pm0.001 ^{+ 0.003 }_{- 0.003 }$
0.40	1.000	1.000

**Table 11:** Unfolded values for 〈*ρ*(*r*)〉, together with statistical and systematic uncertainties for 70 GeV<*p*_T_<100 GeV

*r*	〈*ρ*_*b*_(*r*)〉 [*b*-jets]	〈*ρ*_l_(*r*)〉 [light jets]
0.02	$8.98 \pm0.15 ^{+ 0.55 }_{- 0.54 }$	$12.37 \pm0.38 ^{+0.93}_{- 1.10 }$
0.06	$8.14 \pm0.10 ^{+ 0.17 }_{- 0.17 }$	$5.44 \pm0.16 ^{+ 0.38}_{- 0.39 }$
0.10	$3.80 \pm0.05 ^{+ 0.25 }_{- 0.25 }$	$2.42 \pm0.08 ^{+ 0.18}_{- 0.21 }$
0.14	$1.74 \pm0.03 ^{+ 0.10 }_{- 0.10 }$	$1.52 \pm0.06 ^{+ 0.11}_{- 0.13 }$
0.18	$1.00 \pm0.02 ^{+ 0.03 }_{- 0.03 }$	$0.89 \pm0.04 ^{+ 0.05}_{- 0.05 }$
0.22	$0.66 \pm0.01 ^{+ 0.04 }_{- 0.04 }$	$0.68 \pm0.03 ^{+ 0.05}_{- 0.04 }$
0.26	$0.47 \pm0.01 ^{+ 0.03 }_{- 0.03 }$	$0.45 \pm0.02 ^{+ 0.05}_{- 0.04 }$
0.30	$0.34 \pm0.01 ^{+ 0.03 }_{- 0.03 }$	$0.38 \pm0.02 ^{+ 0.04}_{- 0.04 }$
0.34	$0.26 \pm0.01 ^{+ 0.03 }_{- 0.03 }$	$0.28 \pm0.01 ^{+ 0.03}_{- 0.03 }$
0.38	$0.17 \pm0.00 ^{+ 0.02 }_{- 0.02 }$	$0.18 \pm0.01 ^{+ 0.03}_{- 0.03 }$

**Table 12:** Unfolded values for 〈*Ψ*(*r*)〉, together with statistical and systematic uncertainties for 70 GeV<*p*_T_<100 GeV

*r*	〈*Ψ*_*b*_(*r*)〉 [*b*-jets]	〈*Ψ*_l_(*r*)〉 [light jets]
0.04	$0.359 \pm0.006 ^{+ 0.022 }_{- 0.021 }$	$0.495 \pm0.015 ^{+ 0.037 }_{- 0.042 }$
0.08	$0.678 \pm0.005 ^{+ 0.023 }_{- 0.023 }$	$0.718 \pm0.010 ^{+ 0.032 }_{- 0.037 }$
0.12	$0.827 \pm0.003 ^{+ 0.017 }_{- 0.018 }$	$0.818 \pm0.007 ^{+ 0.019 }_{- 0.021 }$
0.16	$0.891 \pm0.002 ^{+ 0.012 }_{- 0.013 }$	$0.883 \pm0.005 ^{+ 0.012 }_{- 0.014 }$
0.20	$0.928 \pm0.002 ^{+ 0.011 }_{- 0.012 }$	$0.919 \pm0.004 ^{+ 0.010 }_{- 0.011 }$
0.24	$0.954 \pm0.001 ^{+ 0.009 }_{- 0.009 }$	$0.947 \pm0.003 ^{+ 0.008 }_{- 0.009 }$
0.28	$0.972 \pm0.001 ^{+ 0.006 }_{- 0.007 }$	$0.965 \pm0.002 ^{+ 0.007 }_{- 0.008 }$
0.32	$0.984 \pm0.001 ^{+ 0.004 }_{- 0.004 }$	$0.981 \pm0.001 ^{+ 0.004 }_{- 0.005 }$
0.36	$0.993 \pm0.000 ^{+ 0.002 }_{- 0.002 }$	$0.992 \pm0.001 ^{+ 0.002 }_{- 0.002 }$
0.40	1.000	1.000

**Table 13:** Unfolded values for 〈*ρ*(*r*)〉, together with statistical and systematic uncertainties for 100 GeV<*p*_T_<150 GeV

*r*	〈*ρ*_*b*_(*r*)〉 [*b*-jets]	〈*ρ*_l_(*r*)〉 [light jets]
0.02	$11.48 \pm0.20 ^{+ 0.71 }_{- 0.74 }$	$13.89 \pm0.54 ^{+1.60}_{- 1.70 }$
0.06	$7.08 \pm0.11 ^{+ 0.24 }_{- 0.25 }$	$4.68 \pm0.20 ^{+ 0.50}_{- 0.37 }$
0.10	$2.94 \pm0.05 ^{+ 0.23 }_{- 0.23 }$	$2.31 \pm0.11 ^{+ 0.28}_{- 0.29 }$
0.14	$1.37 \pm0.03 ^{+ 0.06 }_{- 0.06 }$	$1.27 \pm0.07 ^{+ 0.09}_{- 0.10 }$
0.18	$0.85 \pm0.02 ^{+ 0.05 }_{- 0.05 }$	$0.74 \pm0.05 ^{+ 0.08}_{- 0.07 }$
0.22	$0.58 \pm0.02 ^{+ 0.04 }_{- 0.03 }$	$0.58 \pm0.05 ^{+ 0.12}_{- 0.10 }$
0.26	$0.39 \pm0.01 ^{+ 0.03 }_{- 0.02 }$	$0.39 \pm0.03 ^{+ 0.08}_{- 0.06 }$
0.30	$0.29 \pm0.01 ^{+ 0.02 }_{- 0.02 }$	$0.31 \pm0.02 ^{+ 0.04}_{- 0.03 }$
0.34	$0.21 \pm0.01 ^{+ 0.02 }_{- 0.02 }$	$0.24 \pm0.01 ^{+ 0.03}_{- 0.04 }$
0.38	$0.14 \pm0.00 ^{+ 0.02 }_{- 0.02 }$	$0.15 \pm0.01 ^{+ 0.02}_{- 0.02 }$

**Table 14:** Unfolded values for 〈*Ψ*(*r*)〉, together with statistical and systematic uncertainties for 100 GeV<*p*_T_<150 GeV

*r*	〈*Ψ*_*b*_(*r*)〉 [*b*-jets]	〈*Ψ*_l_(*r*)〉 [light jets]
0.04	$0.459 \pm0.008 ^{+ 0.028 }_{- 0.030 }$	$0.556 \pm0.022 ^{+ 0.062 }_{- 0.067 }$
0.08	$0.734 \pm0.005 ^{+ 0.019 }_{- 0.020 }$	$0.743 \pm0.014 ^{+ 0.033 }_{- 0.036 }$
0.12	$0.852 \pm0.004 ^{+ 0.013 }_{- 0.012 }$	$0.843 \pm0.010 ^{+ 0.021 }_{- 0.017 }$
0.16	$0.904 \pm0.002 ^{+ 0.010 }_{- 0.010 }$	$0.898 \pm0.007 ^{+ 0.017 }_{- 0.014 }$
0.20	$0.937 \pm0.002 ^{+ 0.008 }_{- 0.008 }$	$0.928 \pm0.005 ^{+ 0.014 }_{- 0.011 }$
0.24	$0.960 \pm0.001 ^{+ 0.006 }_{- 0.006 }$	$0.954 \pm0.003 ^{+ 0.008 }_{- 0.007 }$
0.28	$0.975 \pm0.001 ^{+ 0.005 }_{- 0.005 }$	$0.970 \pm0.002 ^{+ 0.006 }_{- 0.006 }$
0.32	$0.986 \pm0.001 ^{+ 0.003 }_{- 0.003 }$	$0.983 \pm0.001 ^{+ 0.003 }_{- 0.003 }$
0.36	$0.994 \pm0.000 ^{+ 0.001 }_{- 0.001 }$	$0.994 \pm0.001 ^{+ 0.001 }_{- 0.001 }$
0.40	1.000	1.000

These observations are in agreement with the MC calculations, where top-quark pair-production cross sections are implemented using matrix elements calculated to NLO accuracy, which are then supplemented by angular- or transverse momentum-ordered parton showers. Within this context, both MC@NLO and Powheg+Pythia give a good description of the data, as illustrated in Figs. 12–21.

**Fig. 22:**
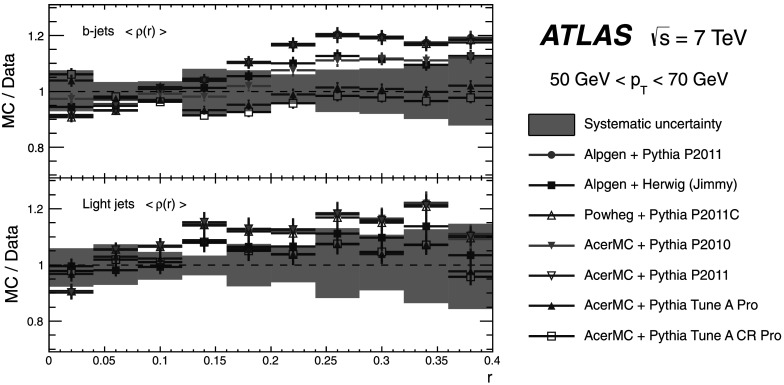
Comparison of the $t\bar{t}$ differential jet shape data for 50 GeV<*p*_T_<70 GeV with several MC event generators. As stated in the text, AcerMC [35] coupled to Pythia [33] with the A Pro and A CR Pro tunes [69, 70] give the best description of the data, while the Perugia 2011 [36] tunes are found to be slightly disfavoured. Alpgen+Jimmy [28, 39] provides an intermediate description.

Comparisons with other MC approaches have been made (see Fig. 22). The Perugia 2011 tune, coupled to Alpgen+Pythia, Powheg+Pythia and AcerMC+Pythia, has been compared to the data, and found to be slightly disfavoured. The AcerMC generator [35] coupled to Pythia for the parton shower and with the Perugia 2010 tune [36] gives a somewhat better description of the data, as does the Alpgen [39] generator coupled to Herwig.

AcerMC coupled to Tune A Pro [69, 70] is found to give the best description of the data within the tunes investigated. Colour reconnection effects, as implemented in Tune A CR Pro [69, 70] have a small impact on this observable, compared to the systematic uncertainties.

Since jet shapes are dependent on the method chosen to match parton showers to the matrix-element calculations and, to a lesser extent, on the fragmentation and underlying-event modelling, the measurements presented here provide valuable inputs to constrain present and future MC models of colour radiation in $t\bar{t}$ final states.

MC generators predict jet shapes to depend on the hard scattering process. MC studies were carried out and it was found that inclusive *b*-jet shapes, obtained from the underlying hard processes $gg\to b\bar {b}$ and *gb*→*gb* with gluon splitting $g\to b\bar{b}$ included in the subsequent parton shower, are wider than those obtained in the $t\bar{t}$ final states. The differences are interpreted as due to the different colour flows in the two different final states i.e. $t\bar{t}$ and inclusive multi-jet production. Similar differences are also found for light-jet shapes, with jets generated in inclusive multi-jet samples being wider than those from *W*-boson decays in top-quark pair-production.

## Summary

The structure of jets in $t\bar{t}$ final states has been studied in both the dilepton and single-lepton modes using the ATLAS detector at the LHC. The first sample proves to be a very clean and copious source of *b*-jets, as the top-quark decays predominantly via *t*→*Wb*. The second is also a clean source of light jets produced in the hadronic decays of one of the *W* bosons in the final state. The differences between the *b*-quark and light-quark jets obtained in this environment have been studied in terms of the differential jet shapes *ρ*(*r*) and integrated jet shapes *Ψ*(*r*). These variables exhibit a marked (mild) dependence on the jet transverse momentum (pseudorapidity).

The results show that the mean value 〈*Ψ*(*r*)〉 is smaller for *b*-jets than for light jets in the region where it is possible to distinguish them, i.e. for low values of the jet internal radius *r*. This means that *b*-jets are broader than light-quark jets, and therefore the cores of light jets have a larger energy density than those of *b*-jets. The jet shapes are well reproduced by current MC generators for both light and *b*-jets.

## Footnotes

1 ATLAS uses a right-handed coordinate system with its origin at the nominal interaction point (IP) in the centre of the detector and the *z*-axis along the beam pipe. The *x*-axis points from the IP to the centre of the LHC ring, and the *y*-axis points upward. Cylindrical coordinates (*r*,*ϕ*) are used in the transverse plane, *ϕ* being the azimuthal angle around the beam pipe. The pseudorapidity is defined in terms of the polar angle *θ* as *η*=−lntan(*θ*/2).

2 The radius in the *η*–*ϕ* space is defined as $\varDelta R = \sqrt{(\varDelta \eta )^{2}+(\varDelta \phi)^{2}}$.

3 The transverse mass is defined as $m_{\mathrm{T}} = \sqrt{2p_{\mathrm {T}}^{\ell}E_{\mathrm{T}}^{\mathrm{miss}}(1-\cos \varDelta \phi_{\ell\nu})}$, where *Δϕ*_*ℓν*_ is the angle in the transverse plane between the selected lepton and the $E_{\mathrm{T}}^{\mathrm{miss}}$ direction.
